# Influenza Virus Drug Resistance: A Time-Sampled Population Genetics Perspective

**DOI:** 10.1371/journal.pgen.1004185

**Published:** 2014-02-27

**Authors:** Matthieu Foll, Yu-Ping Poh, Nicholas Renzette, Anna Ferrer-Admetlla, Claudia Bank, Hyunjin Shim, Anna-Sapfo Malaspinas, Gregory Ewing, Ping Liu, Daniel Wegmann, Daniel R. Caffrey, Konstantin B. Zeldovich, Daniel N. Bolon, Jennifer P. Wang, Timothy F. Kowalik, Celia A. Schiffer, Robert W. Finberg, Jeffrey D. Jensen

**Affiliations:** 1 School of Life Sciences, École Polytechnique Fédérale de Lausanne (EPFL), Lausanne, Switzerland; 2 Swiss Institute of Bioinformatics (SIB), Lausanne, Switzerland; 3 Program in Bioinformatics and Integrative Biology, University of Massachusetts Medical School, Worcester, Massachusetts, United States of America; 4 Department of Microbiology and Physiological Systems, University of Massachusetts Medical School, Worcester, Massachusetts, United States of America; 5 Department of Biology and Biochemistry, University of Fribourg, Fribourg, Switzerland; 6 Center for GeoGenetics, Natural History Museum of Denmark, University of Copenhagen, Copenhagen, Denmark; 7 Department of Medicine, University of Massachusetts Medical School, Worcester, Massachusetts, United States of America; 8 Department of Biochemistry and Molecular Pharmacology, University of Massachusetts Medical School, Worcester, Massachusetts, United States of America; University of Washington, United States of America

## Abstract

The challenge of distinguishing genetic drift from selection remains a central focus of population genetics. Time-sampled data may provide a powerful tool for distinguishing these processes, and we here propose approximate Bayesian, maximum likelihood, and analytical methods for the inference of demography and selection from time course data. Utilizing these novel statistical and computational tools, we evaluate whole-genome datasets of an influenza A H1N1 strain in the presence and absence of oseltamivir (an inhibitor of neuraminidase) collected at thirteen time points. Results reveal a striking consistency amongst the three estimation procedures developed, showing strongly increased selection pressure in the presence of drug treatment. Importantly, these approaches re-identify the known oseltamivir resistance site, successfully validating the approaches used. Enticingly, a number of previously unknown variants have also been identified as being positively selected. Results are interpreted in the light of Fisher's Geometric Model, allowing for a quantification of the increased distance to optimum exerted by the presence of drug, and theoretical predictions regarding the distribution of beneficial fitness effects of contending mutations are empirically tested. Further, given the fit to expectations of the Geometric Model, results suggest the ability to predict certain aspects of viral evolution in response to changing host environments and novel selective pressures.

## Author Summary

In recent years, considerable attention has been given to the evolution of drug resistance in the influenza A H1N1 strain. As a major annual cause of morbidity and mortality, combined with the rapid global spread of drug resistance, influenza remains as one of the most important global health concerns. Our work here focuses on a novel multi-faceted population-genetic approach utilizing unique whole-genome multi-time point experimental datasets in both the presence and absence of drug treatment. In addition, we present novel theoretical results and two newly developed and widely applicable statistical methodologies for utilizing time-sampled data – with a focus on distinguishing the relative contribution of genetic drift from that of positive and purifying selection. Results illustrate the available mutational paths to drug resistance, and offer important insights in to the mode and tempo of adaptation in a viral population.

## Introduction

Influenza A virus (IAV) is an important human pathogen, resulting in approximately 36,000 deaths annually in the United States [1] and eliciting constant concerns regarding the spread of new pandemic strains [2]–[4]. IAV is an eight segment RNA virus that can rapidly evolve owing to a high mutation rate [5], genomic reassortment [6], and stochastic migration of virus from isolated human populations [7] or animal reservoirs [8]. The most common therapies for IAV infections include neuraminidase inhibitors, including the widely used oseltamivir. Oseltamivir was initially designed based on structural information [9], and has been shown to be a competitive inhibitor of the neuramindase active site [10]. Due to the mechanism of action of oseltamivir, it was widely believed that the evolution of drug resistance would decrease fitness of the virus and therefore, be unlikely to be of importance in a clinical setting [11]. However, oseltamivir resistance has been shown to evolve quickly in human hosts [12], [13] and pandemic H1N1 IAV isolates developed resistance to the drug [14]. The most common resistance mutation of H1N1 strains is the H275Y mutation (N2 numbering) which is located near the neuraminidase active site and attenuates oseltamivir binding [10]. The recent rise of oseltamivir resistance in clinical isolates is likely due to the presence of compensatory mutations in the neuraminidase (NA) and hemagglutinin (HA) genes that increase the fitness of the H275Y resistance mutant [15]–[17].

Here, we describe the analysis of IAV populations during the evolution of drug resistance during in vitro growth. This system offers an ideal platform to study the relative effects of genetic drift and selection in evolution, as a target of selection, specifically the H275Y mutation, is known prior to analysis. Further, in vitro growth platforms allow for the control and knowledge of demographic parameters, particularly the severity of population bottlenecks – thus allowing insight into the expected role of genetic drift. Lastly, the high mutation rate and short generation time of IAV allows for adaptation to occur on experimentally tractable time scales.

This experimental set-up allows for an additional benefit – namely, time-sampled whole-genome data. This added temporal dimension provides an important component in the puzzle of disentangling selection and demography – as it becomes possible to utilize analytical results describing the change in frequency [18] and sojourn time [19] of beneficial mutations. Thus, time-sampled data allow the trajectory of any individual allele to be used to better identify the action of natural selection, rather than simply the patterns of genomic variation as utilized by standard single time-point site-frequency spectrum based statistics [20].

Utilizing this experimental approach and the above reasoning, we have tested and developed novel statistical tests of selection for time-sampled population data. We infer effective population size (*N_e_*) in this platform, and develop novel analytical-, maximum likelihood-, and approximate Bayesian -based approaches to determine the contributions of genetic drift and selection in this biological system. Finally, based on this population genetic inference, we demonstrate that IAV development of drug resistance follows the expectations of Fisher's Geometric Model, offering a novel approach to predicting viral evolution in response to changing host environments and novel selective pressures.

## Results and Discussion

**Figure 1:**
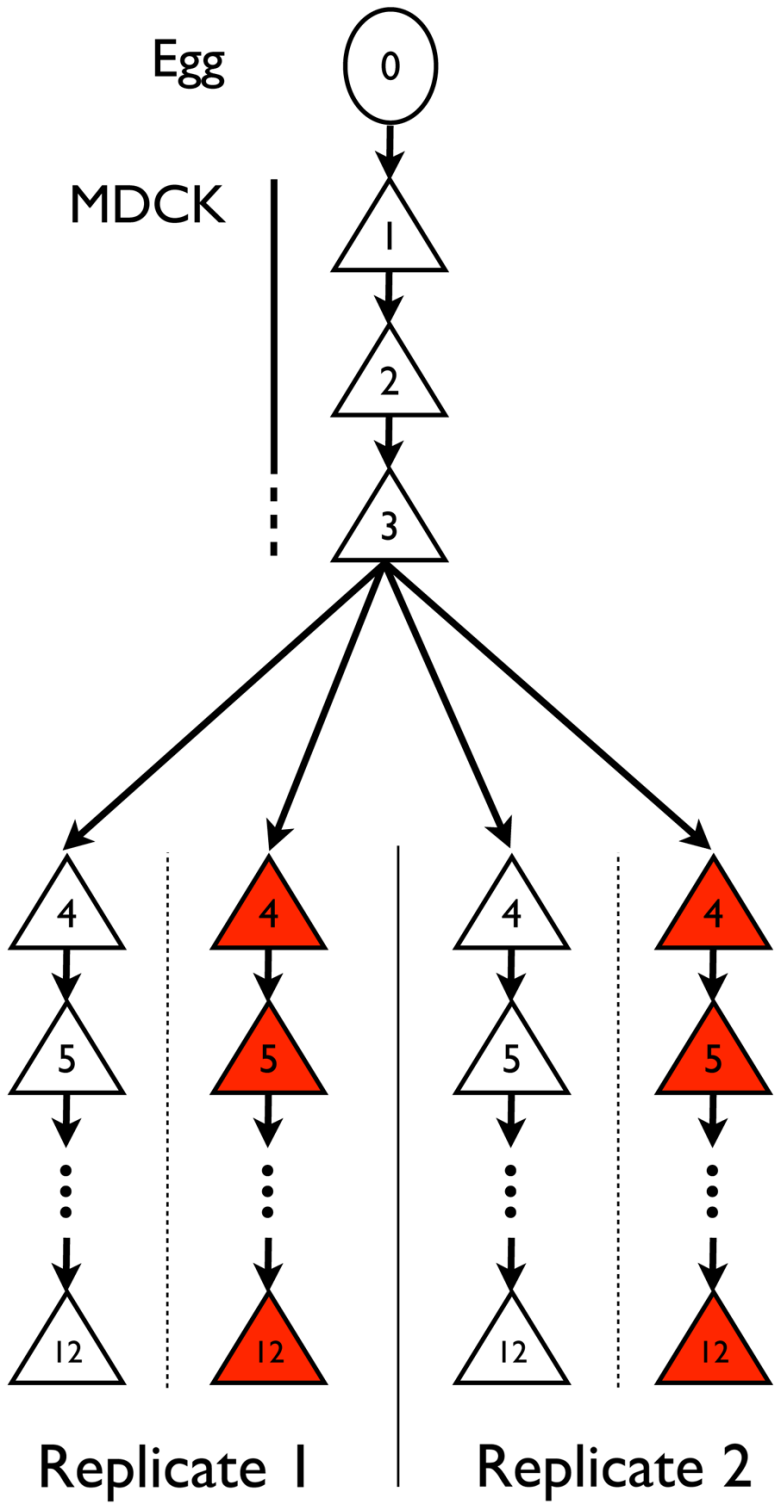
Experimental set-up. IAV was adapted from chicken egg to MDCK cells (passages 1–3) and then serially passaged in MDCK cells in the presence (red) or absence (white) of escalating concentrations of oseltamivir (passages 4–12) in replicate experiments.

Influenza A/Brisbane/59/2007 (H1N1) was initially serially amplified on Madin-Darby canine kidney (MDCK) cells for three passages. The samples were then passaged either in the absence of drug, or in the presence of increasing concentrations of oseltamivir, a neuraminidase inhibitor (Figure 1). At the end of each passage, samples were collected for whole genome high throughput population sequencing providing a high depth of coverage. In addition, biological replicates of the entire experiment were performed and analyzed. We first focus on one of the two experiments in the following results, and then use the replicate as a point of comparison. The genetic diversity calculated as the average expected heterozygosity [21] in each passage was very low, stable throughout the entire experiment, and slightly lower during oseltamivir treatment (*6.2×10^−4^* vs. *4.5×10^−4^*, see Figure S1). The frequency spectra indicated that most single nucleotide polymorphisms (SNPs) were segregating at low frequency and the shape was comparable at P4 and P12 (Figure S2). The number of new mutations accumulated within each passage was limited and very rarely reached high frequencies, in particular in the presence of oseltamivir, suggesting that the viral populations were under severe purifying selection. As a consequence, nearly all observed SNPs were biallelic: over all passages and nucleotides, the frequency of the third allele was 0.02% on average, with a 99% quantile of 0.1%. For this reason, we considered all SNPs as biallelic in our subsequent analyses. Finally, we observed 4 and 7 newly arising mutations reaching a frequency of more than 50% during our experiment in the absence and presence of oseltamivir, respectively.

### Expected impact of drift and ascertainment

**Figure 2:**
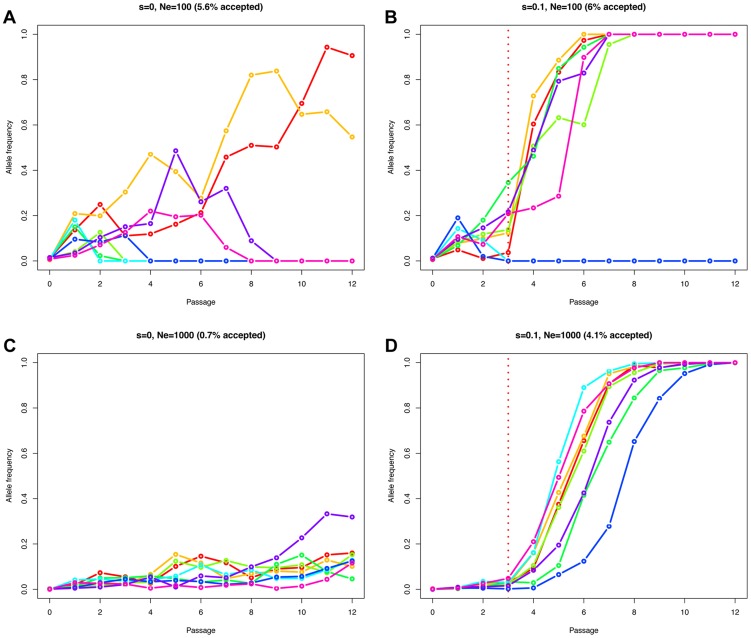
Simulated Wright-Fisher trajectories illustrating the impact of effective population size, selection strength, and ascertainment. We simulated a Wright-Fisher haploid model with selection matching our data set (same number of generations (i.e., 13 per passage), and selection beginning at passage 4). We plot eight randomly drawn trajectories starting from a single mutant and conditioned on reaching at least a frequency of 10% in one of the time points. We used an effective population size of *N_e_ = 100* in A and B, or *N_e_ = 1000* in C and D. The relative fitness of the new mutation was set to *1+s*, with *s = 0* in A and C, or *s = 0.1* in B and D. The fraction of simulations reaching our 10% allele frequency condition is given above each panel in parentheses.

The effective population size *N_e_* determines how efficiently natural selection acts on a population [18]. A beneficial mutation has a greater chance to be successful in large populations compared to small populations, where allele frequency changes are mainly impacted by genetic drift. Therefore, the fate of a beneficial allele is determined by both the effective population size *N_e_* and the selection coefficient *s*. For this reason, observing an allele increasing in frequency cannot be considered as a direct evidence of natural selection. Neutral and deleterious mutations may also increase in frequency, but simply with a lower probability [19]. The problem of distinguishing drift from selection is exacerbated in genome-wide studies, as these low probability events are more likely to occur among the large number of sites considered. Figure 2 illustrates this point, simulating a Wright-Fisher haploid model with selection intensities matching those inferred in the data set. We plot eight randomly drawn trajectories starting from a single mutant and conditioned on reaching at least a frequency of 10% in one of the passages (for *N_e_ = 100* (Figures 2A and 2B) or *N_e_ = 1000* (Figures 2C and 2D), and *s = 0* (Figures 2A and 2C) or *s = 0.1* (Figures 2B and 2D)). In the absence of selection, in populations of low effective size (*N_e_ = 100*), the relative frequency of mutants reaching a frequency of 10% or higher is elevated (5.6% of the simulations) compared to *N_e_ = 1000* (0.7% of the simulations). With selection (*s = 0.1*), these values are nearly unchanged if the population is small (6% vs. 5.6% for *N_e_ = 100*), as low frequency mutants are mostly affected by drift in this scenario. However, values increase dramatically as the effective population size increases (4.1% vs. 0.7% with *N_e_ = 1000*). Finally, the simulated advantageous mutations follow almost deterministic trajectories in large populations (Figure 2D) while drift is still affecting them strongly when *N_e_ = 100* (Figure 2B), eventually leading to the loss of the mutant.

### Estimating *N_e_* and selection

Several methods have been proposed to estimate the effective population size (*N_e_*) from time-sampled data assuming neutrality. Moment-based methods [22]–[25] have the advantage of being computationally efficient as compared to likelihood-based methods and thus can accommodate large genomic data [26]–[29], and can provide similar accuracy when using appropriate estimators [30]. Likelihood methods have the advantage of being able to also take into account the effects of selection, and a handful of methods have recently been proposed to estimate both *N_e_* and selection coefficients [18] from time-sampled data [31]–[33]. However, being based on diffusion approximation [34], they assume large effective population sizes and low selection coefficients. Goldringer and Bataillon [35] proposed to use a moment-based estimator of *N_e_* to reject neutrality based on Wright-Fisher simulations, but currently there is no available method able to co-estimate *N_e_* and *s* in this context. In particular one would like to use the information shared by all loci to estimate *N_e_*, and to estimate *s* at each locus. Here we use *Fs′*, an unbiased estimator of *N_e_* proposed by Jorde and Ryman [30] and extend the idea proposed by Goldringer and Bataillon [35] to also estimate *s* using an Approximate Bayesian Computation (ABC) approach [36] (see Materials and Methods). Our method does not rely on diffusion approximation, is appropriate for small effective population sizes and large selection coefficients, and is computationally efficient to scale with our genomic data.

For both experiments with and without oseltamivir, we respectively estimated *N_e_* to be 226 (99% highest posterior density (HPD) interval: [210;257]) and 176 (99% HPD interval: [117;256]). These low effective population sizes are in line with the values estimated from natural populations in IAV and other viruses [37]–[39]. They can partially be attributed to the severe bottlenecks introduced at each passage, followed by exponential population growth. For comparison, we also calculated the expected effective population sizes as the harmonic mean of census sizes at each generation [40]. We used the estimated census population sizes measured at the beginning of each passage (Table 1) and assumed an exponential population growth to 10^6^ virions during each of the 13 generations. We obtained values of 696 and 737 in experiments with and without oseltamivir, respectively. As expected, this illustrates the strong influence of the bottlenecks despite the very large population sizes assumed (10^6^) at the end of each passage. However, the bottlenecks alone cannot explain the even lower effective population sizes estimated from the full genomic data, though the large variance in burst size (i.e. the number of virions produced per infected cell) [41], [42] is also of relevance [40].

**Table 1:** Bottleneck sizes at each passage.

Passage	1	2	3	4	5	6	7	8	9	10	11	12
Without oseltamivir	48	43	2575	255	85500	8600	27750	4725	37	92	2102	31
With oseltamivir	48	43	2575	255	49250	8600	7300	4800	19	75	2075	42

Population size estimated at the beginning of each passage in the absence or presence of oseltamivir (see Figure 1).

**Figure 3:**
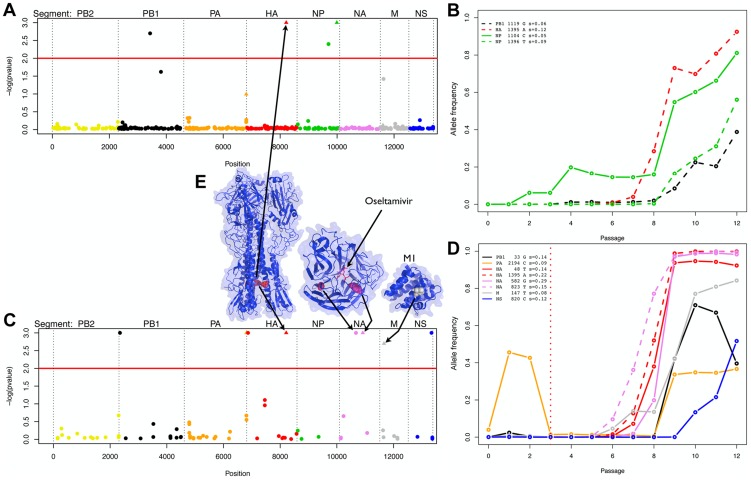
Evidence of positive selection in the H1N1 genome in the absence and presence of oseltamivir. We plot the Bayesian P-values for each SNP in log scale in the absence and presence of oseltamivir in A and C, respectively. The horizontal red lines are genome-wide significance thresholds of *P = 0.01*. The eight segments are separately color-coded, a scheme which is maintained in all panels. Significant nonsynonymous mutations are represented with triangles. We plot the minor allele frequency trajectories of all significant SNPs over the experiment in the absence and presence of oseltamivir in B and D, respectively. The vertical dotted red line indicates the time of the oseltamivir addition (see Figure 1). Trajectories are represented in dashed lines if a second SNP was significant within a given segment. For each significant SNP, the name of the segment, the position of the SNP, the nucleotide increasing in frequency, and the estimated selection coefficients with our *Ne*-based ABC method are indicated in the top left corner of B and D. In E, we represent the 3D structure of the proteins corresponding to the segments coding for membrane proteins (for those with a resolved structure). We indicate the amino acid residues corresponding to the significant mutations with arrows. The SNP locations highlighted on the structures are as follows: HA2 D112N (nonsynonymous), NA G193G (synonymous), H275Y (nonsynonymous), and M1 A41V (nonsynonymous). Although the M segment encodes both the M1 and M2 protein, the significant SNP is only positioned in the coding region of M1. The significant SNP in the NS segment (F116) is synonymous and only positioned in the region coding for the NS2 protein. HA is represented as a trimer, with the significant residue being highlighted (red) in each monomer, though one residue is slightly obscured due to being buried in the protein complex. NA and M1 are represented as monomers, and NA is shown with a bound molecule of oseltamivir.

We then obtained posterior distributions of *s* for all contending mutations (i.e., mutations that were not lost by drift and are segregating in the population in at least one time point). Neutrality was rejected when the posterior density interval of *s* excluded zero (i.e., 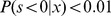), defining Bayesian ‘p-values’ [43]. These p-values are plotted for all sites in the genome in Figure 3A and 3C. Note that there are fewer sites in the presence of oseltamivir (82 vs. 405, see Figure 3C and 3A) as fewer time points match the criteria defining contending mutations. We plot the trajectories corresponding to the significant sites in the absence and presence of drug respectively in Figure 3B and 3D. Despite the reduced data size, more sites are found to be under selection in the presence of oseltamivir (8, representing almost 10% of the sites considered; versus 4, representing less than 1% of the sites in the absence of drug), and having larger selection coefficients (0.15 on average vs. 0.08). In addition, an HA mutation (HA 1395 encoding a D112N mutation in HA2) was positively selected in both the absence and presence of drug, suggesting that it likely represents a tissue culture adaptation. However, the mutation was nonetheless more strongly beneficial in the presence of drug (*s* = 0.22 vs. *s* = 0.12, see Figure 3B and 3D).

**Figure 4:**
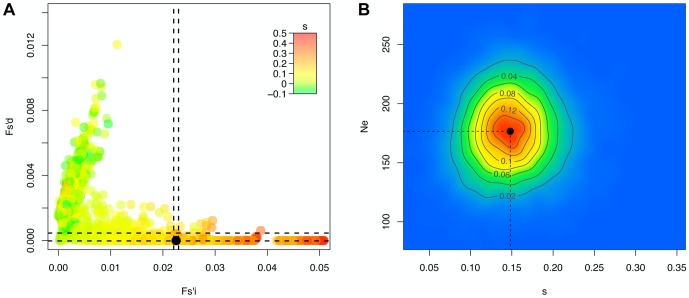
*Ne*-based Approximate Bayesian Computation for the H275Y resistance mutation in the presence of oseltamivir. For 10'000 simulated trajectories (out of the 100'000 simulations performed), we plot in A the values of the statistics *Fsi′* and *Fsd′* with colors corresponding to the selection coefficients *s*, as well as the values calculated for the real trajectory of the H275Y oseltamivir resistance mutation in black. We indicate the region corresponding to the best 1% retained simulations with a dashed line, and plot the corresponding two-dimensional posterior distribution for *s* and *N_e_* in B.

The known H275Y resistance mutation [10] located on the NA protein at position 823 in the RNA sequence goes rapidly to fixation in the presence of oseltamivir (Figure 3D) with a point estimate of *s = 0.15*. The corresponding posterior distribution is represented in Figure 4 along with the ABC correlation plot. The separate correlation between *s* and the two statistics *Fsd′_i_* and *Fsi′_i_* is also shown in Figure S3. In the presence of drug, NA and HA are the two segments containing the mutations with the highest selection coefficients (0.20 on average, compared to 0.11 for the other segments). This finding is in accordance with recent results showing that mutations in HA compensate for the deleterious effect (low growth capacity) of H275Y [17]. We extracted from the NCBI Influenza Virus Resource database [44] the recent changes in allele frequency of the 12 candidate sites under selection in natural populations of H1N1 (Figure S4). Two out of 12 candidates increased rapidly in frequency in the past five years. As previously reported [14], H275Y started to increase in frequency in the 2007/2008 influenza season and almost reached fixation in 2009/2010 (90.1%). A synonymous mutation in segment NS at position 820 (F116) also increased very rapidly in frequency in 2005/2006 (92.6%), decreasing again to less than 12% in the following years to finally reach fixation in 2009/2010.

We randomly simulated 1000 pseudo-observed data sets to cross validate our ABC approach with parameters inspired by the drug-treated experiment. We used the same number of loci with selection coefficients *s* taken from the obtained posterior mean, *N_e_ = 176*, a sample size of *1000* and initial allele frequencies of *1/N_e_*. For each simulated replicate, we estimated *N_e_* and *s* using our proposed ABC approach (see Materials and Methods). This simulation represents a “worst-case” scenario, as the impact of genetic drift is strong and allele frequencies are skewed. Despite this, we found that the accuracy is generally very good (Figure S5A and B). We note that as the trajectories start from a single mutant, it is difficult to distinguish negative selection from neutrality, both generally leading to a rapid loss of the mutant and to a wide posterior for *s* with a mean around zero. The same phenomenon is also observed for beneficial mutations, but at a lower frequency. The problem vanishes when the initial allele frequency is increased, or in larger populations (data not shown).

Finally, we evaluated whether our model based on constant effective population size is robust to the series of bottlenecks and population expansions induced at each passage in the experiment (see Table 1). Ewens [45] showed that in a population with a size changing cyclically over time, the probability of fixation of an allele is approximately the same as in a population of constant size with N_e_ = N*, where N* is the harmonic mean of the population sizes at each generation, which is also the effective population size of the fluctuating population. To evaluate this finding in the context of our estimation procedure, we simulated 1000 additional data sets with varying population sizes. In order to match the drug-treated experiment, 9 passages of 13 generations each were simulated, with exponential population growth from *N = 23* to *N = 10^6^* (see Figure 1). The founding population size (*N = 23*) was chosen as it results in a harmonic mean of *N_e_ = 176* for the 13 generations (i.e., the empirically estimated value from the experiment). Here again, this corresponds to a “worst-case” scenario where the bottleneck at each passage is extremely strong (see Table 1 for the true data) and population expansion very rapid. We found that our ABC procedure based on a constant effective population size indeed accurately estimates *N_e_* and *s* (Figure S5C and D) - with *N_e_* being slightly downwardly biased (estimated to 167 on average), and large selection coefficients are very slightly upwardly biased.

### Effect of genetic linkage

Linkage between selected and neutral sites can confound inference when estimating genome wide selection coefficients. Further, the frequency of homologous recombination in the IAV genome is still debated [46]. If absent, we expect to observe strong effects of genetic hitchhiking [47], where linked sites should increase in frequency together with selected sites due to physical linkage. However, the very low genetic diversity in our populations limits this phenomenon, and we identified at most two selected sites within the same segment. We also note that the initial allele frequencies of the sites under selection are very low in all cases (Table 2) and below our detection threshold (0.17%) in 11 out of the 12 cases. This suggests that selection is primarily acting on de novo mutations rather than on standing variation in our experiment.

**Table 2:** Estimated selection coefficients.

	Segment	Position	Protein	Sequential numbering	Other numbering	Allele	Initial frequency	Final frequency	*Ne*-ABC *s* estimates (99% HPDIs)	Malaspinas *et al.* [32] *s* estimates
Without	PB1	1119	PB1	L373		G	0.01%	38.8%	0.06 (0.01;0.11)	0.04
oseltamivir	**HA**	**1395**	HA	D455N	D112N (HA2)	**A**	**0.03%**	**92.5%**	**0.12 (0.05;0.19)**	**0.08**
	NP	1104	NP	N368		C	0.02%	81,1%	0.05 (0.00;0.11)	0.04
	**NP**	**1396**	NP	L466F		**T**	**0.03%**	**56.1%**	**0.09 (0.03;0.15)**	**0.06**
With	*PB1*	*33*	PB1	K11		*G*	*0.03%*	*39.6%*	*0.14 (0.06;0.25)*	*0.03*
oseltamivir	PA	2194	PA	Noncoding		C	1.4%	36.7%	0.09 (0.02;0.17)	0.01
	HA	48	HA	L6		T	0.1%	92.3%	0.14 (0.06;0.27)	0.18
	**HA**	**1395**	HA	D455N	D112N (HA2)	**A**	**0.06%**	**99.9%**	**0.22 (0.08;0.34)**	**0.27**
	NA	582	NA	T194		G	0.02%	98.3%	0.29 (0.15;0.45)	0.41
	**NA**	**823**	NA	H275Y	H274Y (N2)	**T**	**0.04%**	**99.5%**	**0.15 (0.06;0.24)**	**0.20**
	**M**	**147**	M1	A41V		**T**	**0.04%**	**84.2%**	**0.08 (0.01;0.15)**	**0.07**
	NS	820	NS2	F116		C	0.03%	51.7%	0.12 (0.04;0.20)	0.09

Comparison of *N_e_*-ABC and Malaspinas *et al.* [32] estimates of *s* for the significant trajectories under selection. Bold indicates nonsynonymous mutations and italic indicates a poor fit to our Wright-Fisher model. We indicate the nucleotide corresponding to the minor allele, with its initial frequency at the beginning of the experiment in the absence of oseltamivir, or at passage 4 when drug treatment began (see Figure 1). For the *Ne*-ABC method, we give the 99% highest posterior density intervals (HPDIs) in brackets.

Only two trajectories in each experiment were identified as having a poor fit to the assumed Wright-Fisher model, and are shown in Figure S6. Figure S7 also shows the ABC correlation and posterior plots for one of these cases, where one can see the inability of the model to generate simulations similar to the observed data. Clonal interference (i.e., the competition of simultaneously segregating beneficial mutations) has recently been proposed to play an important role in influenza evolution [48] and could explain such patterns, in which, in the absence of recombination, an initially steep trajectory becomes halted or even reversed by the appearance of another more beneficial mutation (as in Figure S6A). However, given the small effective population sizes in this experiment, it is not surprising that we do not observe more such trajectories, as the probability that multiple contending beneficial mutations are present at a given point is small [49]. Integrating the potential of clonal interference into the methodology developed here will be the subject of future study.

### Replicated experiment

The experiment was replicated starting at passage 4 (Figure 1), and analyzed with our *N_e_*-based ABC method. We plot the Bayesian p-values for all sites in the genome in Figure S8A and S8C and the trajectories corresponding to the significant selected sites, in the absence and presence of drug, in Figure S8B and S8D, respectively. More details are given on selected sites in Table S1. Consistent with the first experiment, more sites are identified as being under selection in the presence of oseltamivir (6 vs. 2). The H275Y resistance mutation appears only at passage 9 and also increases very rapidly in frequency in the presence of oseltamivir (Figure S8D) with an even higher selection coefficient *s = 0.27*. Interestingly, like in the first experiment, one nonsynonymous HA mutation (position 1211, encoding a N50K mutation in HA2) is under strong selection in both the absence and presence of oseltamivir, and is located only 184 base pairs from the one identified in the first experiment (position 1395, encoding a D112N mutation in HA2). Similarly, a nonsynonymous mutation in segment M at position 92 (encoding an E23Q mutation of the M1 protein) is also under selection (*s = 0.06*), where in the first experiment, one was identified at position 147 (encoding an A41V mutation of the M1 protein, *s = 0.08*).

### Likelihood and coalescent based estimation

**Figure 5:**
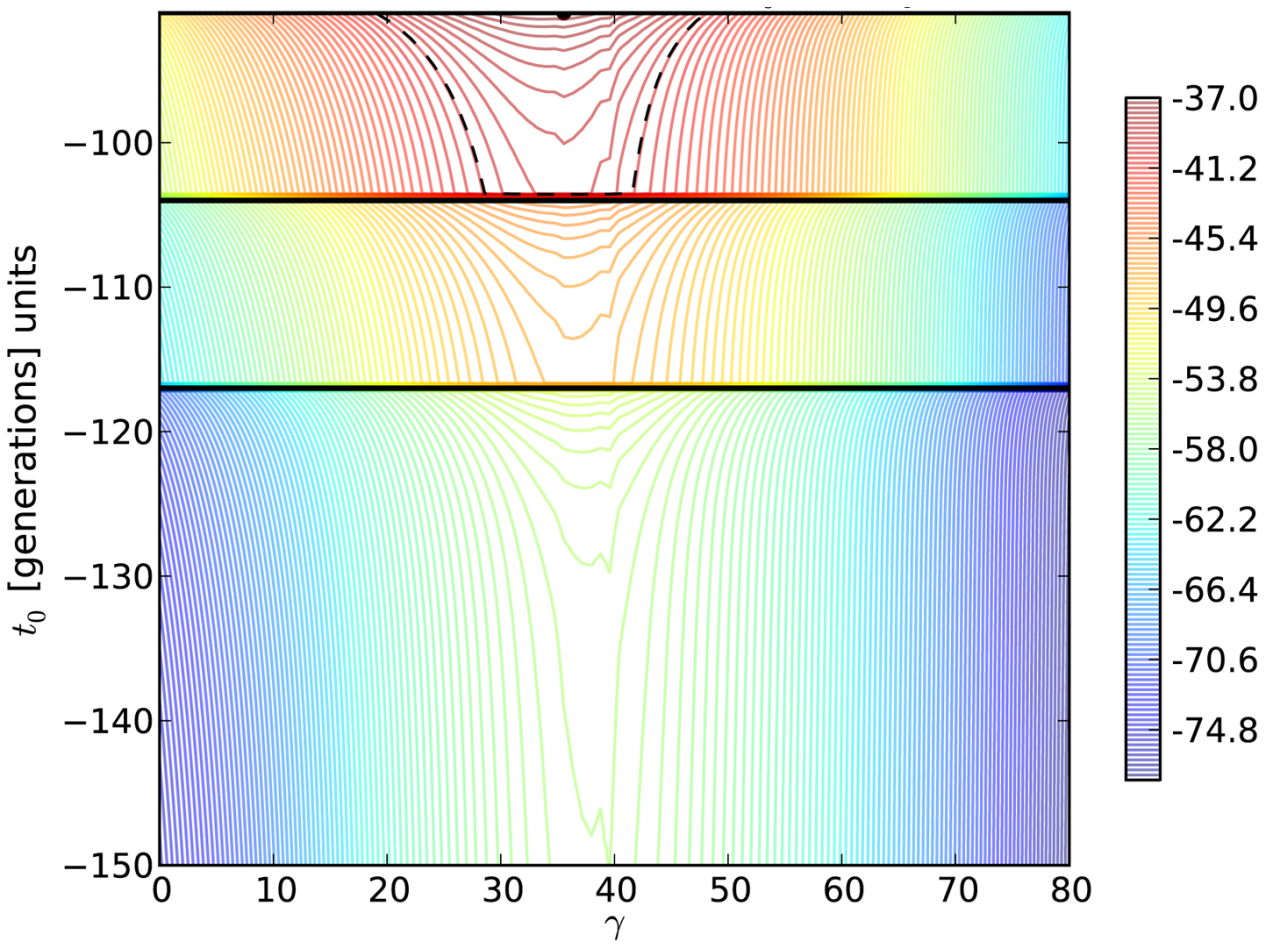
Log-likelihood contours for the H275Y resistance mutation in the presence of oseltamivir. We plot the two-dimensional likelihood surface for the selection parameter *γ = N_e_^.^s* (x-axis) and the allele age *t_0_* (y-axis) in generations, with generation zero representing the end of the experiment. Horizontal black lines represent sampling times. The colors indicate the value of the log-likelihood, with red being the highest and blue being the lowest. The maximum likelihood is shown as a black dot, and the log likelihood was lowered from its peak by 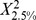/2 = 2.996, to construct a 95% confidence (likelihood) region for the parameters (dashed black line).

As a matter of comparison, for each significant trajectory identified using our *N_e_*-based ABC method, we applied a diffusion approximation likelihood-based method [32] which we here extend to a haploid model. For the H275Y mutation, the two-dimensional likelihood surface for *γ = N_e_^.^s* and the age of the mutation (*t_0_*) is shown in Figure 5. The selection coefficients obtained are mostly consistent between the two methods and are given in Table 2. They tend to be different when the trajectories have an unexpected behavior under the Wright-Fisher model, like the synonymous PB1 mutation at position 33 (K11), also identified as having a poor fit to our model. In this case the behavior of the two methods is hard to interpret. Likelihood-based methods in general should be more accurate than ABC methods, which reduce the whole data in to a few summary statistics. However, as stated previously, the diffusion approximation made to calculate the likelihood in this method is not appropriate for high selection coefficients and low effective population sizes (i.e., it is appropriate for *N_e_s*∼1). As expected, this method tends to over-estimate *s* compared to our ABC method when *s* is large. Indeed, in this approximation, drift is a slow process and very large allele frequencies changes as we observe can only be explained by excessively large values of *s*. Additionally, there is currently no available likelihood method that can integrate the information shared by all sites to infer *N_e_*, and estimating *N_e_* from a single site is particularly inefficient [32]. For this reason, and to have a fair comparison, we did not attempt to estimate *N_e_* here, but fixed it to the value estimated above via the ABC approach. Finally, we note that this class of method is computationally intensive and cannot practically be applied to whole-genome datasets.

**Figure 6:**
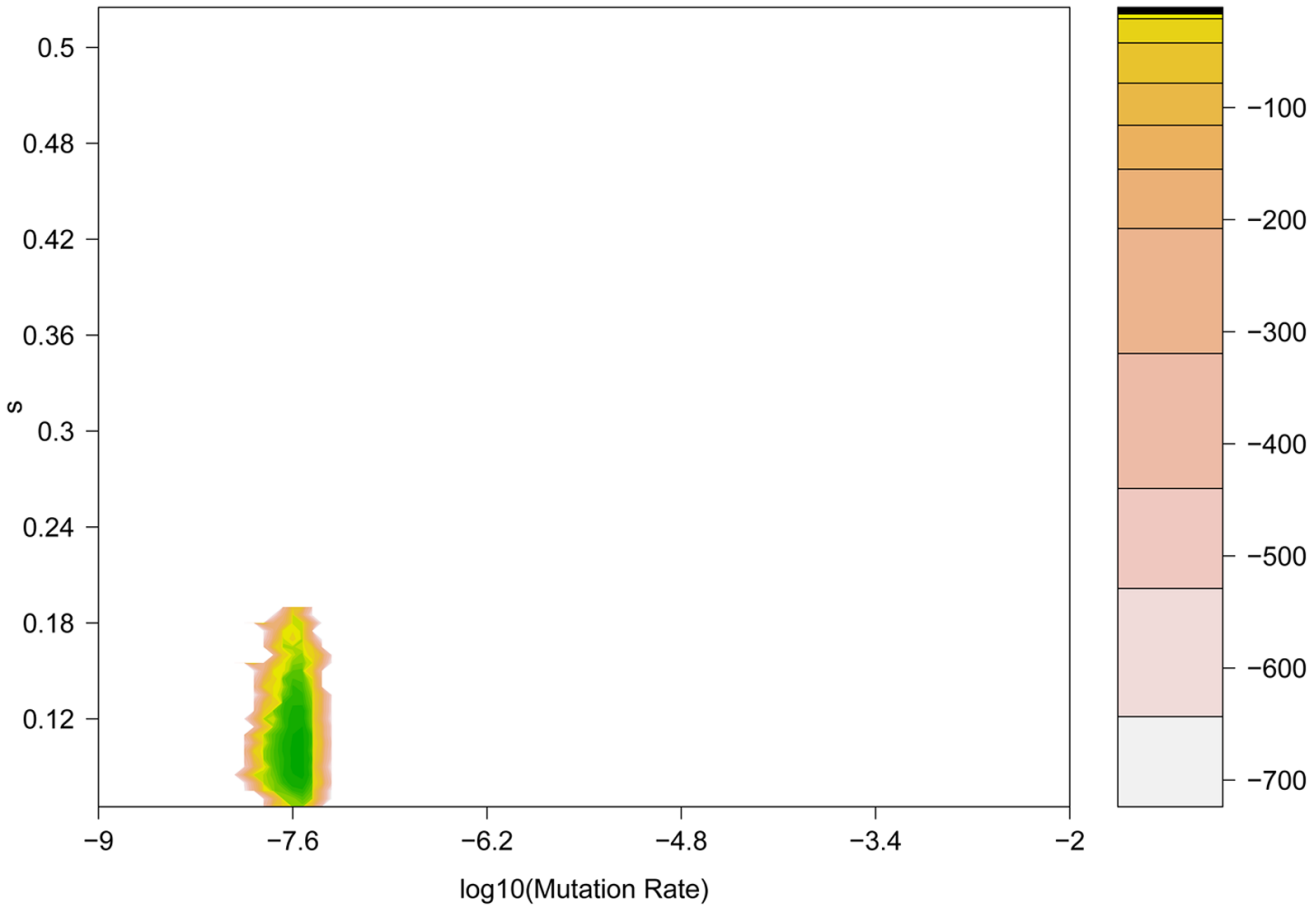
Coalescent-based maximum likelihood surface for the H275Y resistance mutation in the presence of oseltamivir. Two-dimensional likelihood surface for the selection coefficient (*s*) (y-axis) and the mutation rate *μ* (x-axis). The graph is colored according to the value of the log-likelihood (displayed in the embedded legend), where green indicates the highest probability and yellow the lowest.

In addition, we have developed a new coalescent-based method that explicitly models the known demography of our experimental populations for comparison (Table 1). This approach has the additional advantage of incorporating the genetic diversity linked to the beneficial mutation into the estimation procedure, allowing us to estimate the mutation rate and refine estimates of *s*. Figure 6 shows the combination of selection coefficient (*s* = 0.12) and mutation rate (*μ*≈10^−7^) with the highest likelihood for the H275Y mutation. Interestingly, the estimation obtained with these simulations accounting for the true demography is consistent with our *N_e_*-based ABC method, which gives a posterior mean for the selection coefficient of *s = 0.15* (Table 2 and Figure 4).

It is noteworthy that the three methods here developed and applied to this data are complementary, and thus have been used jointly. Our *N_e_*-based ABC approach is computationally efficient, can be applied to large genomic datasets, and does not rely on diffusion approximation. It provides both estimates of *N_e_* using information from the whole genome, and posterior distributions for *s* at each individual site. Using these results, it is next interesting to utilize the likelihood-based method, as it can be more accurate for small *s* (or in cases where *N_e_* is large), as it uses the full data rather than summary statistics. Additionally, it can estimate the age of the beneficial allele, which can be of interest in some cases. Being more computationally intensive, one can apply it on the candidate sites identified by the *N_e_*-ABC method, and even take advantage of the estimated *N_e_* that it provides. Finally, our new coalescent method is a promising first attempt to estimate *s* not only using a single allele frequency trajectory, but the whole sequence linked to it. It is also computationally very intensive and can be used on top candidate sites to refine the posterior distributions obtained from the *N_e_*-based ABC method.

### Fisher's Geometric Model and distance from optimum

Further utilizing these estimated per-site *N_e_*-based selection coefficients, and in order to contextualize these results, we utilize the framework of Fisher's Geometric Model (FGM). The FGM [50] predicts that environmental challenges increase the distance between the current phenotype and the phenotypic optimum, thereby allowing for more and stronger beneficial mutations.

Here, the oseltamivir environment represents a novel (and challenging) environment, which is expected to result in a shift of the optimum away from the location of the current population. This is reflected both in a higher maximum beneficial selection coefficient (0.288 vs. 0.117) and in a higher mean beneficial selection coefficient (0.026, bootstrap bias-corrected and accelerated [51] 95% confidence interval (CI): [0.017; 0.039] vs. 0.016, 95% CI [0.015; 0.017]), obtained from the point estimates obtained using the *N_e_*-based ABC approach - indicating that the optimum may be indeed further away from the current phenotype in the drug as compared with the no-drug environment.

In order to study the distribution of fitness effects (DFE) observed, and to quantify the distance to the phenotypic optimum in each environment, we chose three different biologically relevant distributions to be fitted to the data. Since we were particularly interested in the distance to the phenotypic optimum, we used the displaced-gamma distribution proposed by Martin and Lenormand [52], which results in an approximately beta-shaped DFE of beneficial mutations [53], and allows for a direct estimate of the distance to the optimum under the FGM. Secondly, we used a half-normal distribution, which is predicted from the FGM, when the optimum is (infinitely) far away. Third, we used the generalized Pareto distribution (GPD). Given that beneficial mutations are so rare that they represent the tail of the full DFE, the GPD allows for the estimation of the most likely extreme value domain [54]. In other words, the resulting estimate of the shape parameter *κ* yields information on whether the DFE is bounded (i.e., the full DFE belongs to the Weibull domain), whether its tail is exponential-like (Gumbel domain), or whether the tail is heavier than exponential (Frechet domain). In terms of the underlying biology, this is a very important question: for example, a bounded DFE would indicate that mutations cannot exceed a certain effect size, whereas, on the contrary, a heavy-tailed DFE would suggest that mutational effect sizes are highly unpredictable. Supporting arguments have been made for an exponential tail being the most biologically reasonable [55], and related studies have suggested an exponential distributions of fitness effects (e.g., [56], [57]). However, there also exists empirical evidence for bounded tails of the beneficial DFE (e.g., [58], [59]).

Of note, our data consist not of the full distribution of newly arising beneficial mutations, but of the fraction of those beneficials that survive drift and are segregating in the population for at least a short time (so-called “contending beneficial mutations” [60], [61]). Barrett *et al.* [60] pointed out that this distribution arises as the full distribution of new mutations weighted by the (approximate) probability *1-exp(-2s)* that a mutation with selection coefficient *s* survives drift. The resulting probability densities for the three tested distributions are noted in the Materials and Methods.

**Figure 7:**
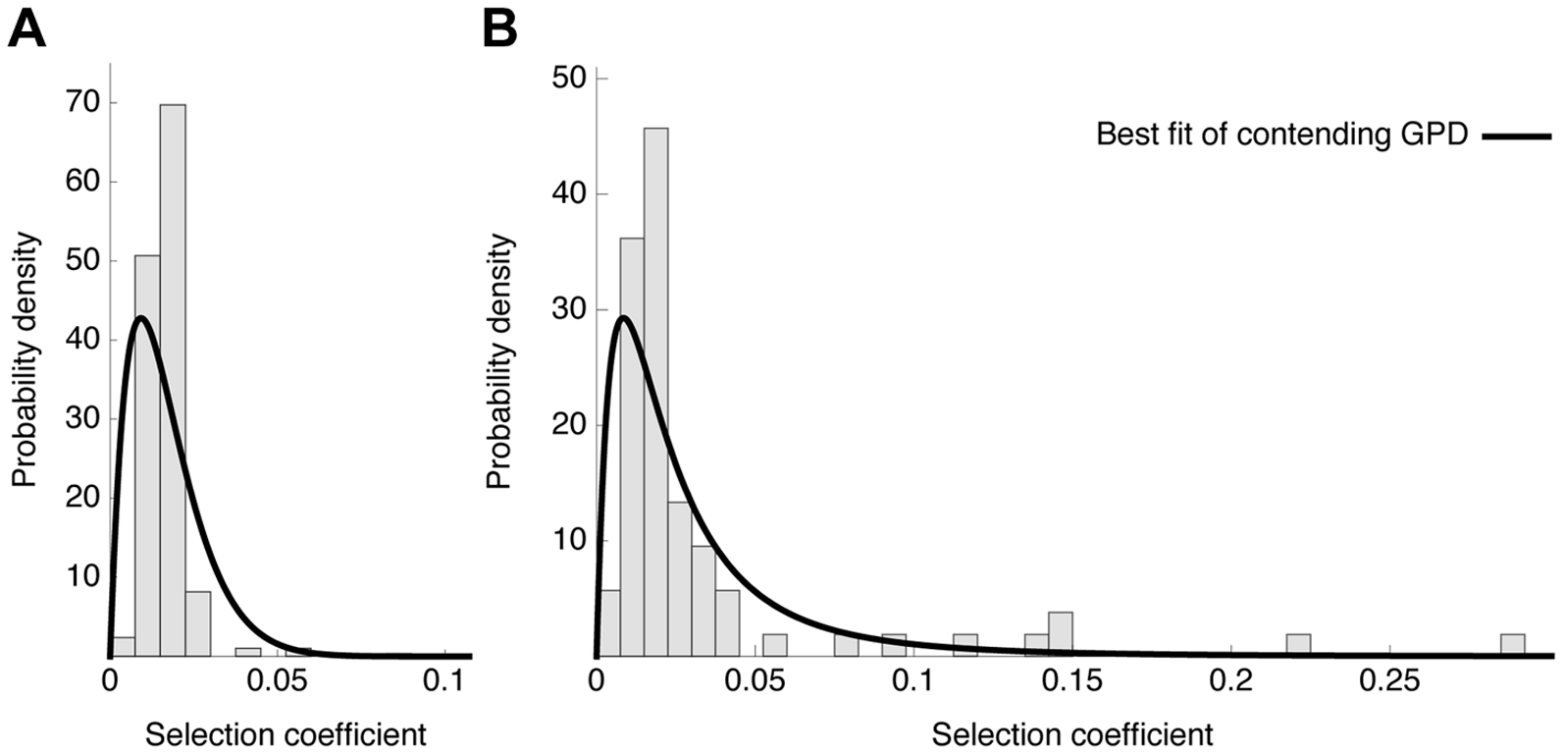
Distribution of fitness effects. Observed histograms of fitness effects of contending beneficial mutations and the best fit of a contending generalized Pareto distribution (solid lines) in the absence (A) or presence (B) of oseltamivir. The heavy tail of the DFE in the presence of the drug is clearly visible.

**Figure 8:**
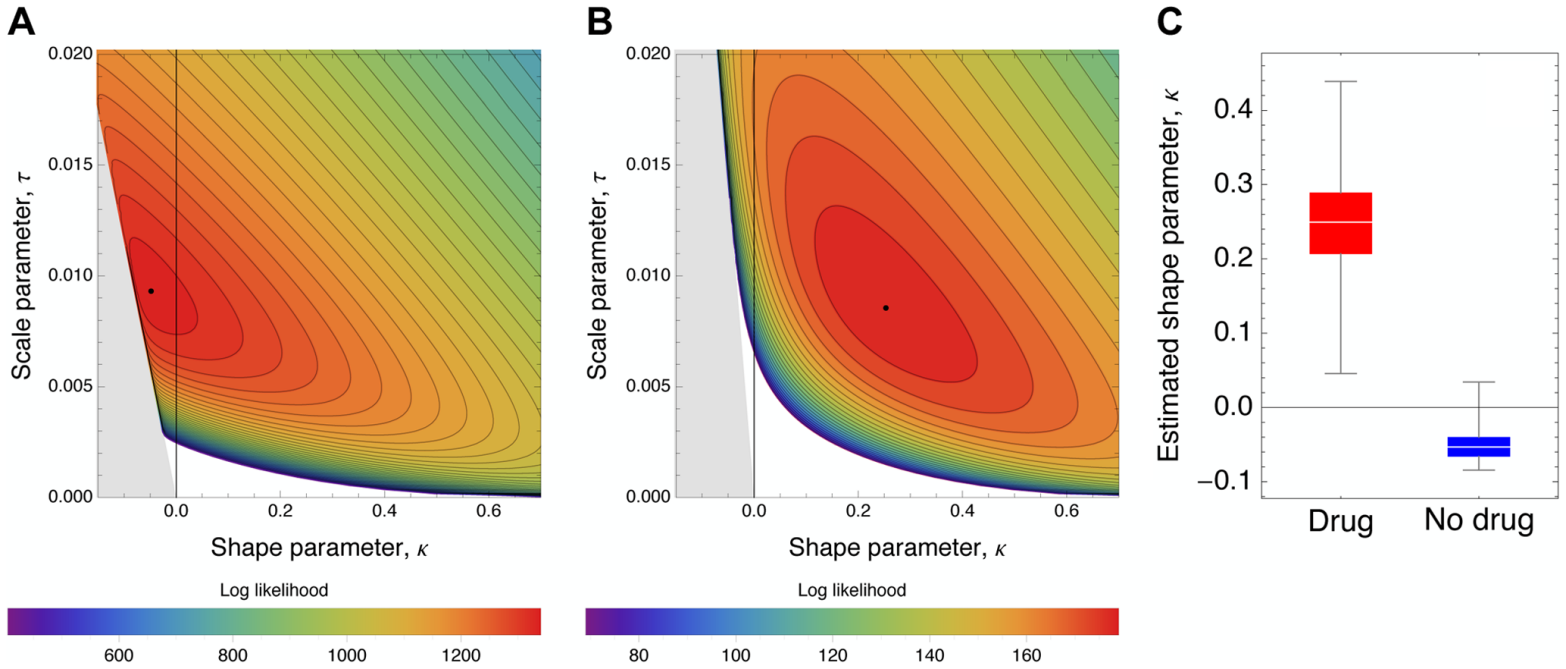
Likelihood surfaces for the contending generalized Pareto distribution. Likelihood surfaces of the fit of a contending generalized Pareto distribution in the absence (A) or presence (B) of oseltamivir. The shape parameter *κ* (on the x-axis) determines the domain of attraction. The maximum likelihood estimate (indicated by a black dot) lies in the Weibull domain (*κ<0*) in the absence of the drug, indicating a bounded distribution of fitness effects, whereas it lies in the Frechet domain (*κ>0*) in the presence of the drug, indicating an underlying heavy-tailed distribution of fitness effects. Black lines represent 25 (A) and 5 (B) orders of log likelihood, respectively. The gray area represents inaccessible parameter space, because the lower bound of the estimated DFE cannot be lower than the maximum observed selection coefficient. We also show the distribution of the shape parameter κ for the GPD distribution (C).

We fitted all three resulting contending distributions to the data by numerically maximizing the log likelihood using a weighted bootstrap approach [62]. All results are reported in Table 3 and Figures 7 and 8.

**Table 3:** Maximum likelihood estimates of the distribution of fitness effects.

		With oseltamivir		Without oseltamivir	
Distribution	k^1^	Log(L) (95% CI)^2^	*s_0_* (95% CI)^3^	Log(L) (95% CI)	*s_0_* (95% CI)
GPD	2	180.0 [154.7,199.1]	∞	1343.3 [1301.5,1374.0]	0.17 [0.12,0.76]
Beta	2	158.8 [122.1,190.0]	∞	1343.4 [1301.4,1374.1]	0.18 [0.12,1]
Half-normal	1	110.5 [69.3,154.7]	∞	1375.6 [1274.4,1429.7]	∞

Theoretical distributions of fitness effects fitted to the data.

1 Number of parameters.

2 Maximized value of the log-likelihood function with 95% confidence intervals.

3 Maximum possible effect-size.

In the absence of oseltamivir, the half-normal distribution yields the highest median log likelihood (cf. Table 3, and Figure S9A), but the bootstrap estimates show a large variance (also owing to the lower flexibility afforded by the single parameter). Hence, the outcome is sensitive to potential measurement error, or sampling bias. In fact, the 95% confidence intervals of the bootstrap estimates of both the GPD and the beta distribution lie within that of the half-normal, indicating that they both may represent equally good summaries of the true distribution. In the presence of oseltamivir, the GPD clearly provides the best fit, with both the half-normal and the scaled beta distribution reaching generally lower log likelihoods (cf. Figure S9B).

The generally good fit of the GPD provides support for the assumption that the beneficial portion of the DFE represents a tail distribution. If this condition is met, the GPD is expected to be the most flexible among the three tested distributions, because it can account for all possible tail shapes. Because we observe only the contending distributions in the present study, the tail of the distribution becomes even more important, and it must be noted that the tails of both the beta scaled and the half-normal distributions studied here are both contained within the GPD.

After establishing that the GPD yielded a good fit to the observed data, we interpreted the estimated shape parameter *κ* that determines the extreme value domain of the underlying DFE (cf. Figure 8). In the absence of oseltamivir, we observe *κ = −0.0532* (95% CI: −0.0786, −0.0075), indicating that the full DFE belongs to the Weibull domain of attraction (Figure 8A). Hence, it has a right-truncated tail, and we estimated the maximum possible mutational effect as *d = 0.17* (95% CI: 0.12, 0.76). We can compare this result with the estimate of the distance to the optimum obtained from the scaled beta distribution. With *s_0_ = 0.18* (95% CI: 0.12, 1), this estimate agrees nicely with that from the GPD. In terms of the underlying biology, this indicates that there is remaining potential for adaptation also in the absence of oseltamivir, but that the maximum possible effect size does not differ greatly from the observed maximum selection coefficient (*s = 0.117*).

The pattern looks very different in the presence of oseltamivir. Here, the estimated shape parameter is *κ = 0.24* (95% CI: 0.14, 0.38), clearly indicating a heavy-tailed DFE that belongs to the Frechet domain of attraction (Figure 8B). Supporting this finding, it was not possible to obtain any reasonable estimates of the distance to the optimum under the scaled beta distribution. This support for a heavy-tailed distribution is also consistent with recent results examining the DFE in populations of yeast that have been subjected to extreme environmental conditions [63].

This finding suggests that the potential for adaptation in the drug environment is indeed much higher than the highest observed selection coefficient, and that mutational effect sizes will be difficult to predict under strong adaptive challenges. In particular, upon a longer run of the experiment (e.g., over the course of time in natural populations) even stronger beneficial mutations than those identified in the present experiment could be expected (however, it is noteworthy that the H275Y mutation appears to be re-identified across multiple different experiments, as discussed below). In comparison, there is only little potential for adaptation in the no-drug environment. However, the optimum is still far as compared with other examples from the literature [64], which could indicate ongoing adaptation to the MDCK cells. We note that we do not explicitly model experimental errors in our analyses, as this would require several replicated experiments [54], [56]. However, the heavy tail of the DFE in the presence of oseltamivir (Figure 7B) may indeed be influenced by such factors, and this result should thus be interpreted with caution.

### Biological implications

Lastly, we attempted to interpret these results in light of the known biology of influenza (Figure 3E). The NA mutation H275Y is a well-characterized oseltamivir-resistance mutation and has been shown to alter the hydrophobic pocket of the NA active site, thereby reducing affinity for drug [10], [65]–[69]. Thus, re-identification of this substitution aids to validate the results. Further, A41V (encoded by a C147T SNP) and E23Q (encoded by a G92C SNP) substitutions of the M1 protein were identified in the first and second experiments, respectively. The location of these mutations (helices 3 and 2) are not overlapping with regions important for RNA or membrane binding, which facilitate virion assembly and maintenance of virion integrity. In addition, the location of either residue does not appear to be important for forming extended sheets of M1 protein, as proposed previously [66]. Therefore, the role of these mutations in viral fitness in the presence of oseltamivir may be related to additional roles of the M1 protein. An intriguing possibility is that interactions between M1 and the NA cytoplasmic tail important during virion budding [70]–[72] are altered by the H275Y mutation and are compensated for by additional mutations in the N-terminal region of M1 [73]. Two adaptive substitutions were also observed in the HA2 peptide during growth in the presence and absence of oseltamivir, with the D112N (encoded by a G1395A SNP) and N50K (encoded by a C1211A SNP) substitutions observed in the first and second replicates, respectively. Interestingly, the trajectories of the substitutions in the presence of drug appear to be strongly correlated with the rise of the H275Y mutation (see Figures 3 and S7). The combined results from the two replicates in the presence and absence of oseltamivir show that these loci are positively selected during tissue culture adaptation, and may epistatically interact with the H275Y allele. The D112N mutation has been well characterized in other influenza strains and is conserved across all HA serotypes. This substitution has been shown to cause a rise in the pH of the HA conformational change and HA-induced endosome and viral membrane fusion [68], [74], a process critical for IAV infectivity [75]. Further, the D50K mutation is located in a region known as the HR1 heptad repeat, interrupting the repeat pattern with a polar-to-charged residue substitution. The HR1 repeat regions form coiled coils that undergo conformational changes in low pH conditions and likewise promote endosomal membrane fusion during IAV infection [76]. Alterations of endosomal membrane fusion mediated by mutations in HA2 are a known mechanism for tissue culture adaptation [77], [78], but interactions with a drug resistance allele has not been described previously. In total, while the results are not sufficient to confirm this hypothesis, the combined results from replicate 1 and replicate 2 suggest that epistatic interactions between M1 and NA, and possibly HA2, may be important during the selection of drug resistance in IAV populations.

### Conclusions

As a major annual cause of morbidity and mortality, influenza virus infections remain one of the most important global health concerns. Foremost amongst the challenges in treating this virus has been its ability to adaptively respond to drug treatment, with oseltamivir resistance spreading globally during the 2007–2008 and 2008–2009 influenza seasons. In order to evaluate the viral adaptive response to oseltamivir, we have here developed a multi-faceted population genetics approach based upon an unparalleled dataset consisting of whole-genome multi-time point experimental data both in the presence and absence of treatment. Utilizing novel approximate Bayesian, likelihood-based, and analytical results, we identify a handful of known and unknown positively selected variants, and quantify the distance from phenotypic optimum imposed by oseltamivir. These results not only confirm a number of theoretical expectations arising from Fisher's Geometric Model and its extensions, but also clearly illustrate the ease by which resistance may be evolved against neuraminidase inhibitors. We finally note that the robust methodologies developed here can be widely applied to time-sampled data from not only experimental but also natural populations (Figure S4), allowing for the utilization of a temporal dimension that is highly informative for identifying the recent action of positive selection.

## Materials and Methods

### Data generation and bioinformatics

Influenza A virus A/Brisbane/59/2007 (H1N1) from chicken egg allantoic fluid (NIH Biodefense and Emerging Infectious Research Resources Repository NIAID, NIH; NR-12282; lot 58550257) was serially passaged in Madin-Darby canine kidney (MDCK) cells (Figure 1). This strain has the following genome size: segment 1 (PB2) 2314 nucleotides (nts), segment 2 (PB1) 2302 nts, segment 3 (PA) 2203 nts, segment 4 (HA) 1776 nts, segment 5 (NP) 1497 nts, segment 6 (NA) 1427 nts, segment 7 (M1/2) 1006 nts, segment 8 (NS1/2) 870 nts. MDCK cells were maintained in Eagle's minimal essential medium (MEM) with 10% fetal bovine serum (Hyclone) and 2 mM penicillin/streptomycin. Viral infections were performed in influenza virus growth medium as described [79] and lasted for 72 hours. Virus was continually passaged on cells to prevent any freeze-thaw cycles and the amount of virus to initiate a passage and the virus at the end of each passage were subsequently empirically determined via plaque assays using standard techniques. These values were used to determine the bottleneck size (Table 1), MOIs (Table S2), magnitude of population expansion, and number of doublings associated with each passage. The number of doublings was used to determine the number of generations per passage – averaging to 13 generations per passage throughout the experiment, in both the no drug and drug-treated populations.

For passages indicated in Figure 1, oseltamivir was added at increasing concentrations and two independent experimental trajectories were performed. The initial concentration of oseltamivir was equal to the ED_50_, the concentration of drug that reduced viral plaque numbers to 50% of a no drug control. The initial ED_50_ was 0.1 uM (Table S2), indicating that the starting virus was very sensitive to oseltamivir. The next passage was performed in the presence of 4× ED_50_. Subsequent passages were performed by doubling the concentration of oseltamivir if 50% cytopathic effect (CPE) was observed (i.e., the cytopathic effect in the cells from the previous passage). If 50% CPE was not observed, the dose of oseltamivir was reduced to a concentration that lead to the observation of 50% CPE. Oseltamivir carboxylate (RO0640802-002; lot 91ST1126/1) was obtained from Roche (F. Hoffmann-La Roche Ltd, Basel, Switzerland). Concentration of oseltamivir at each passage can be found in Table S2.

Cell-free virus was obtained at each passage by spinning down supernatant 72 hour post-infection, and subjected to whole genome pooled population sequencing. Viral RNA was purified using the RNeasy 96 Kit (Qiagen, Gaithersburg, MD). SuperScript III First-Strand Synthesis Supermix (Life Technologies, Grand Island, NY) and primers that bind the 3′ end of all IAV segments were used for reverse transcription. The cDNA was then amplified in a single multiplex reaction to amplify all segments of the genome with near equal efficiency, using primers that have been described previously. The amplified product was sheared to a size range of 300–600 base pairs with Fragmentase from New England Biolabs (Ipswitch, MA) using the procedure recommended by the supplier. DNA was then end repaired, A-tailed, and ligated to Illumina-compatible adapters containing 6-mer barcode sequences. The products were size selected by using 0.8× AMPure XP beads (Agencourt, Beverly, MA), collecting supernatant and treating with 1.6× beads, and eluting DNA with ddH_2_0. After size selection, DNA was amplified with Illumina PE PCR primers, quantified and combined into libraries for sequencing on the HiSeq2000 platform. All sequences used in this study were generated from 100 base pair reads. All sequence data is publicly available for download at http://bib.umassmed.edu/influenza/.

In addition to viral samples, an RNA error control was generated from a cloned influenza A/Brisbane/59/2007 (H1N1) NA gene segment. The cloned segment was used as a template in a T7 transcription reaction to make a pool of control RNA, which was processed and sequenced in parallel with the viral samples. Sequence data from the RNA control showed that 95% of erroneous SNP calls could be eliminated by excluding low frequency (<0.17%) SNPs. Sequence reads were aligned to Influenza A/Brisbane/59/2007 reference genome (Genbank accessions CY030232, CY031391, CY058484–CY058486, CY058488–CY058489, CY058491) using the BLAST alignment algorithm.

Reads were filtered to eliminate those with Phred quality score <20 across the read, and the minimum length of the mappable read >20 nucleotides. The coverage was high with a median over all passages of 56667 (Table S3 and Figure S10), with 90% of reads mapping. We excluded all sites having coverage lower than 100 and we randomly down-sampled all sites having a coverage higher than 1'000, to 1'000 in order to estimate allele frequencies from allele counts. As we almost only observed biallelic SNPs (see results), for each site we kept only the two alleles having the highest frequencies over all passages, and called the minor allele as the one having the lowest frequency at passage 0. In all subsequent analyses concerning the effect of oseltamivir, we omitted the first three passages of pre-drug treatment.

### *N_e_*-based ABC estimation

The observed data 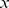 consists of allele frequency trajectories measured at *L* loci: 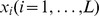. We have one parameter *N_e_* shared by all loci in the genome, and *L* locus-specific selection coefficients 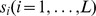 that we would like to infer. In a Bayesian setting, we want to estimate the joint posterior distribution
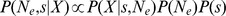
The likelihood 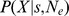 can be calculated numerically in some cases but it relies on approximation and is computationally very intensive (see below). For this reason, we propose a likelihood-free approach based on Approximate Bayesian Computation (ABC) [36], [80]. This class of methods is based on Monte Carlo simulations, which are compared to observed data using summary statistics. In our model, locus-specific summary statistics capable of estimating *s* per locus are needed, as are statistics utilizing information from all loci jointly in order to infer *N_e_*. However, the standard ABC algorithm is not usable in such cases, as the probability of obtaining one simulation with a good match to the observed data for all *L* loci simultaneously rapidly tends to 0 as *L* increases. Recently, Bazin *et al.* [80] proposed a new algorithm to overcome this difficulty where the problem is split in to two steps (Algorithm 2), and we adapt their general solution to our problem here.

First we note that we can decompose the posterior as
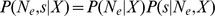
Using conditional independence (see Appendix in [80]), the joint density has the factorization
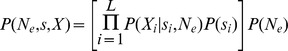
and the marginal density is
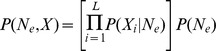
where
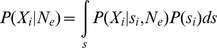
Dividing these two densities we have
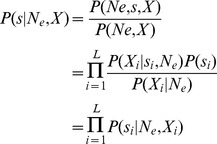
Finally the joint posterior can be factorized as
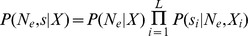
and if focused on a particular locus *i*, we find
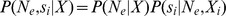
This justifies the need for both locus-specific summary statistics 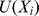 and summary statistics that are a function of all loci together 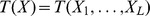, and we approximate the posterior as
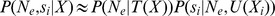



The general algorithm to sample from this posterior adapted from Algorithm 2 in Bazin *et al.* [80] can be written as:

Step 1. Obtain an approximation of the density
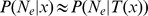



Step 2. For locus *i = 1* to *i = L*

For *k* = 1 to *k* = *N* iterations:

- Sample 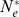 from 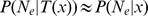 generated in step 1.
- Sample 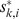 from the prior distribution 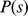.
- Simulate data 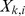 (at locus *i* only) from 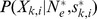.
- Compute 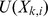.

Condition on 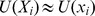 using ABC to obtain a sample of observation 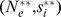 from an approximation to 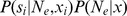.

As noted by Bazin *et al.* [80], if we write the data as 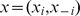 (where the subscript *−i* indicates all data except that from locus *i*), 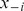 is only used once (in the first step) but the second step re-uses the data 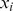 a second time. The algorithm samples from the correct posterior distribution only if we use 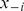 instead of the full data 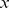 in the first step. Otherwise it involves an approximation, which is valid if we assume that
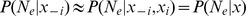
The rationale behind this approximation is that when the number of loci *L* is large, any given locus *i* provides a negligible contribution to the information about *N_e_* (see below).

We now give the details of the two steps of this algorithm. In the original algorithm [80], the first step is also achieved using ABC. In our case, we take advantage of having an existing moment-based estimator of *N_e_* available. This also allows us to avoid the assumption of independence between loci by using a Bayesian block bootstrap approach (see below). We define 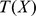 as a single statistic given by Jorde and Ryman *Fs′* unbiased estimator of *N_e_* [30]. For all sites, we calculated *Fs′* between consecutive pairs of time points when the minor allele frequency has reached at least 2% in one observation [30] as:
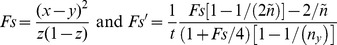
where *x* and *y* are the minor allele frequencies at the two time points separated by *t* generations, 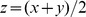, and 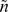 is the harmonic mean of the sample size 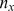 and 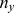 at the two time points. We keep only the sites where we could obtain at least two values of *Fs′* in order to average them over time, and thus obtain an estimator for each site of the genome used in step 2 below. Note that in the experiment involving oseltamivir, as only passages 3 to 12 were considered, a small number of sites matched this criterion. We also averaged *Fs′* values over sites in order to obtain a genome-wide estimator as in [30]. All *Fs′* values were converted to *N_e_* assuming *t* = 13 generations per passage and *N_e_ = 1/Fs′*.

A segment-based Bayesian block bootstrap approach [81], [82] was used to obtain a distribution for 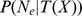, as we cannot assume the independence of sites owing to linkage disequilibrium. More specifically, as multiple virus infections in cells can lead to segment reassortment [6], we grouped sites by segment in order to obtain an estimator for each segment, and randomly resampled segments with replacement 10'000 times using a Dirichlet prior [81]. We also checked that the approximation 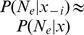 is valid in our case by repeating step 1 for each segment, where all sites in the considered segment are excluded in order to account for linkage. We found that the posteriors are very similar to that obtained using the full data (Figure S11). *N_e_* was only slightly increased when we excluded the segments carrying the highest number of beneficial mutations in the presence of oseltamivir (respectively estimated to *N_e_* = 195 and *N_e_* = 199 when we excluded HA and NA, compared to *N_e_* = 176 when considering all segments).

The second step of our method uses the effective size estimated in the first step as a prior distribution to estimate selection coefficients (*s*) at each site in the genome using an ABC approach [36], [80]. For each site, 100'000 time-sampled trajectories were simulated using a Wright-Fisher haploid model with selection [18] with three conditions: *(i)* the trajectories started at the same minor allele frequency observed at this site [35], *(ii)* the trajectories match the same criteria used on the real data to calculate *Fs′*, and *(iii)* the samples are simulated as a binomial sampling using the per-site sample sizes. For each trajectory, we randomly sample *N_e_* from the 10'000 posterior samples obtained in the first step. The relative fitness of the beneficial allele was set to *1+s*, and we used a uniform prior for *s* between −0.1 and 0.5, as we always consider the minor allele. In the presence of selection, allele frequency trajectories are expected to be directional, whereas drift introduces random variance. Being a measure of variance, *Fs′* does not incorporate information about the direction of allele frequency changes. To integrate this into our estimation procedure, we decomposed *Fs′* at a given site in to two statistics: *Fsd′* and *Fsi′* calculated respectively between pairs of time points, where the allele considered is decreasing and increasing in frequency, such that *Fs′ = Fsd′+Fsi′*. Using notations of our algorithm presented above, this means that at each locus *i* we take 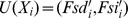. We retained the best 1% of the 100'000 simulations based on the Euclidian distance between observed and simulated *Fsd′* and *Fsi′* statistics in order to obtain posterior distributions and means for *s* using a rejection ABC algorithm [36].

We selected candidate trajectories based on the posterior distribution obtained for *s* at each site: we define Bayesian ‘p-values’ for *s* as 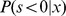 and consider a trajectory to be ‘significant at level *p*’ if it's equal-tailed *100(1−p)%* posterior interval excludes zero [43]. We also performed a cross-validation procedure for the ABC method: we randomly simulated 1000 pseudo-observed data sets with parameters inspired by the drug-treated experiment, with both fixed and varying *N_e_* (see Results). For each simulated replicated, we estimated *N_e_* and *s* using our proposed ABC approach.

We finally identified trajectories for which the Wright-Fisher model has a poor fit based on the Euclidian distance between our simulations and the data. Using the cross-validation procedure, we obtained the null distribution of this distance under the true model, and used the 99% quantile of the distribution as a threshold to detect trajectories in our data not fitting the model.

### Likelihood-based estimation

The outlier trajectories were selected with the *N_e_*-based ABC method when the probability of being beneficial was larger than 99%, and used in a likelihood-based method [32] for comparison. The time-serial method of Malaspinas *et al.* [32] is an extension of Bollback *et al.* [31] to infer the selection coefficient, the effective population size, and additionally the allele age from temporal allele frequency data. A Hidden Markov Model (HMM) is used to model the allele frequency trajectory and an approximate transition density is applied to compute the likelihood. Here, the method is modified to fit a haploid model. The diffusion process approximating the Wright-Fisher haploid model with selection is defined by [34], [83]:
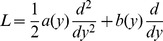


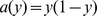


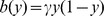
where *y* is the density of allele frequency at time *t* in units of *N_e_* generations and *γ = N_e_·s*.

The state space of the HMM are the population allele frequencies denoted by *z_i_ = i/N_e_* for 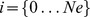. The diffusion process defining the transition probabilities is approximated with a one-step process, which only allows the transition to occur between adjacent states (*z_i_* to *z_i_*_−1_, *z_i_* and *z_i_*_+1_). The infinitesimal generator *Q* for the one-step process is a tridiagonal *(N_e_+1)×(N_e_+1)* matrix:
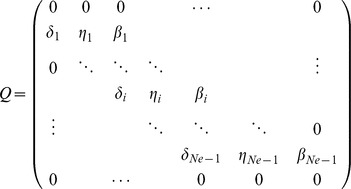
where *β_i_* denotes the rate of jumping from *z_i_* to *z_i_*_+1_, *δ_i_* the rate of jumping from *z_i_* to *z_i_*_−1_, and *η_i_* = *β_i_*+*δ_i_* such that *1-η_i_* is the rate of staying in state *z_i_*.

When the approximation of the one-step process is applied, the solution for the system can be obtained as:
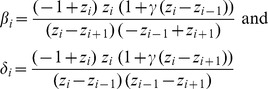



The effective population size *N_e_* was set at 176 in the presence of oseltamivir and at 226 in the absence of drug as inferred by the *N_e_*-based ABC method (see Results). For each candidate trajectory, the maximum likelihood of *γ* and allele age *t_0_* was obtained using the Nelder-Mead optimization algorithm [84], where the search range of *γ* was set to (0,80). For the allele age *t_0_*, the time point of the first appearance of the derived allele was set to be the upper bound of the search range.

### Coalescent-based estimation

We generated allele frequency trajectories for the beneficial allele under a range of selection coefficients *s* between 0 and 0.5 with 0.005 increments, performing 10'000 simulations for each value. These frequency trajectories were produced using a Wright-Fisher haploid model with selection as described above. For replicate 1 of the drug-treated H1N1 strain, we calculated the population size at each generation using the estimated census population sizes measured at the beginning of each passage (Table 1). Further, we assume exponential population growth for 13 generations during each passage, reaching a final population of 10^6^ virions. We accepted those trajectories presenting a difference between the derived allele frequency of observed and simulated data lower than *ε = 0.10* at all time points (from passage 3 to passage 12).

The accepted trajectories were used to run the simulation software *msms* [*85*] under a demographic model resembling the experimental data (twelve consecutive passages with selection starting at passage 4) (Figure 1 and Table 1). *msms* is a coalescent simulation program that incorporates time-sampled data [86] and conditional coalescent on frequency trajectories. *msms* was used to obtain the tree length starting at passage 3 (drug free) and finishing at the end of the experiment (passage 12). We used a search range for the mutation rate *μ* between 10^−9^ and 10^−2^ and computed the probability of observing the total number of segregating sites present in the whole genome of H1N1 for replicate 1 (9666 segregating sites) given the total tree length multiplied by the mutation rate using binomial sampling. Finally, we computed the probability of observing the real data given the simulated by integrating over all possible genealogies *G*:
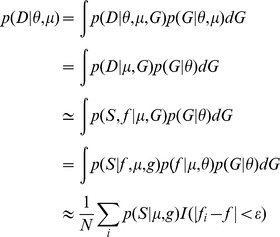
where *D* refers to the total number of segregating sites (*S*) and the derived allele frequency of the beneficial allele (*f*). *θ* represents the demographic model and *μ* the mutation rate. *f_i_* and *f* respectively represent the frequency of the simulated and observed beneficial allele, and *I* the indicator function.

### Estimation of the shape of the distribution of fitness effect

*Mathematica 9.0* was used to fit the distributions using numerical maximization of the log-likelihoods of the data under the given distribution. The probability density functions resulting from weighting the original distribution with the fixation probability of a beneficial mutation, *1-e^−2s^*, and subsequent normalization were performed as follows:

Contending half-normal distribution:
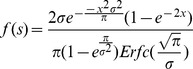
where *Erfc(x)* is the complementary error function, for 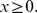


Scaled beta distribution:
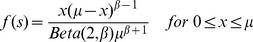
where Beta(a,b) is the Euler beta function.

Contending generalized Pareto distribution:
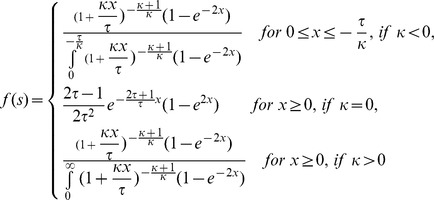



In order to evaluate the confidence in the estimated parameters, a weighted likelihood bootstrap was performed as described by Newton and Raftery [62]. 1000 weighted likelihoods were obtained according to Equation 1 therein, with weights being randomly drawn from a uniform Dirichlet distribution. For the scaled beta distribution, the boundary parameter *s_0_* was limited to values smaller or equal to 1 to ensure successful maximization, and if the estimated MLE yielded *s_0_ = 1*, we concluded that no distance to the optimum could be obtained – in fact, in all tested cases in the presence of oseltamivir the likelihood appeared to increase monotonically as *s_0_* approached infinity (results not shown).

### Structural analysis

Residues that mutated during the course of the experiments were highlighted on the structures of HA, NA and M1. The structure for HA of Influenza A/Brisbane/59/2007 has been generated via molecular modeling [87] and was used in this study. Closely related NA (PDB 3CL0) and M1 (PDB 1EA3) structures were also used. Images were generated in PyMol.

## Supporting Information

Figure S1 Genetic diversity of H1N1 throughout the experiment. The genetic diversity measured as the average expected heterozygosity in passages 4 to 12 of our experiment in the absence (dotted line) or presence (dashed line) of oseltamivir.
(PDF)

Figure S2 Site frequency spectra (SFS) of H1N1 populations during the experiment. The SFS at passages 4 (A and B) and 12 (C and D) is shown in the absence (A and C) and presence (C and D) of oseltamivir.
(PDF)

Figure S3 ABC correlation plot. The correlation between the simulated selection coefficients *s* and the two statistics (*Fs′i* (A) and *Fs′d* (B)) used in our ABC method. Note that Figure 4A is showing the same correlation using colors.
(PDF)

Figure S4 Frequency of identified beneficial mutations in natural populations. The allele frequency in natural populations from the NCBI Influenza Virus Resource database for the significant mutations identified to be under selection - plotted between years 2004 and 2010.
(PDF)

Figure S5 Cross validation of our *Ne*-based ABC method. The true vs. the estimated values of *N_e_* (A and C) and *s* (B and D) for the 1000 simulated data used to validate our ABC procedure. We used similar parameters to our real data: sample size = 1000, *Ne = 176* and initial allele frequencies of *1/Ne*. We simulated a population of constant size (A and B) or experiencing recurrent bottleneck (*N* = 23) followed by exponential growth (up to *N* = 10^6^) mimicking our experiment (C and D). Error bars in B and D represent the 10% and 90% quantiles over the 1000 replicates. The red dot in A and C and the red line in B and D indicate the true value.
(PDF)

Figure S6 SNPs with poor fit to the Wright-Fisher model. The minor allele frequency trajectories of all SNPs identified as not fitting the Wright-Fisher model in the absence and presence of oseltamivir respectively in A and B. The horizontal dotted red line indicates the start of oseltamivir treatment (see Figure 1). Trajectories are represented in dashed lines if a second SNP was significant within the same segment. For each SNP, the name of the segment, the position of the SNP, the nucleotide increasing in frequency, and the estimated selection coefficients with our *Ne*-based ABC method are indicated in the top left corner of A and B.
(PDF)

Figure S7 *Ne*-based Approximate Bayesian Computation for SNP PB1 33 (K11). For 10'000 simulated trajectories (out of the 100'000 simulations performed), we plot in A the values of the statistics *Fs′_i_* and *Fs′_d_* with colors corresponding to the selection coefficients *s*, as well as the values calculated for the real trajectory of the poor fitting PB1 33 (K11) mutation (see Figure S5) in black. We indicate the region corresponding to the best 1% retained simulations with a dashed line, and we plot the corresponding two-dimensional posterior distribution for *s* and *N_e_* in B. We clearly see in A the inability of the model to generate simulations near the observed data, with the black dot being outside the retained regions defined by the dashed lines.
(PDF)

Figure S8 Evidence of positive selection in the H1N1 genome in the absence and presence of oseltamivir for replicated data. We plot the Bayesian P-values of each SNP in log scale in the absence and presence of oseltamivir in A and C, respectively. The horizontal red lines are genome-wide significance thresholds of *P = 0.01*. The eight segments are separately color-coded, a scheme which is maintained in all panels and in Figure 3. Significant nonsynonymous mutations are represented with triangles. We plot the minor allele frequency trajectories of all significant SNPs over the replicated experiment in the absence and presence of oseltamivir respectively in B and D. The horizontal dotted red line indicates the start of oseltamivir treatment (see Figure 1). All colors and line styles match those in Figure 1. Trajectories are represented as dashed lines when a second SNP was significant in a segment, and dotted lines for a third SNP. For each significant SNP, the name of the segment, the position of the SNP, the nucleotide increasing in frequency, and the estimated selection coefficients with our *Ne*-based ABC method are indicated in the top left corner of B and D.
(PDF)

Figure S9 Maximum likelihood for the DFE fit. The boxplots show the distribution of the maximum log-likelihoods obtained from 1000 samples of a weighted likelihood bootstrap in the absence and presence of oseltamivir in A and B, respectively.
(PDF)

Figure S10 Genome wide sequence coverage data for samples used in this study. The coverage in log scale for our four experiments at passages 0, 6 and 12 (see Figure 1).
(PDF)

Figure S11 Estimated *N_e_* when excluding segments. The posterior distribution obtained for *N_e_* using step 1 of our ABC algorithm when excluding each segment one by one in the absence (A) and presence (B) of oseltamivir. Segment colors match those in Figure 3 and S8.
(PDF)

Table S1 Estimated selection coefficients for the replicate experiment. Comparison of *N_e_*-ABC and Malaspinas *et al.* [32] estimates of *s* for the significant trajectories under selection for the replicate experiment. Bold indicates nonsynonymous mutations. We indicate the nucleotide corresponding to the minor allele, with its initial frequency at the beginning of the experiment in the absence of oseltamivir, or at passage 4 when drug treatment began (see Figure 1). For the *Ne*-ABC method, we give the 99% highest posterior density intervals (HPDIs) in brackets.
(PDF)

Table S2 Viral passaging and drug concentration data for samples used in this study.
(PDF)

Table S3 Genome wide sequence coverage data for samples used in this study.
(PDF)
