# Supplementary figures and images for: Influenza Virus Drug Resistance: A Time-Sampled Population Genetics Perspective
Source: PLoS Genet. 2014 Feb 27;10(2):e1004185. doi: 10.1371/journal.pgen.1004185 (PMC3937227; doi:10.1371/journal.pgen.1004185)

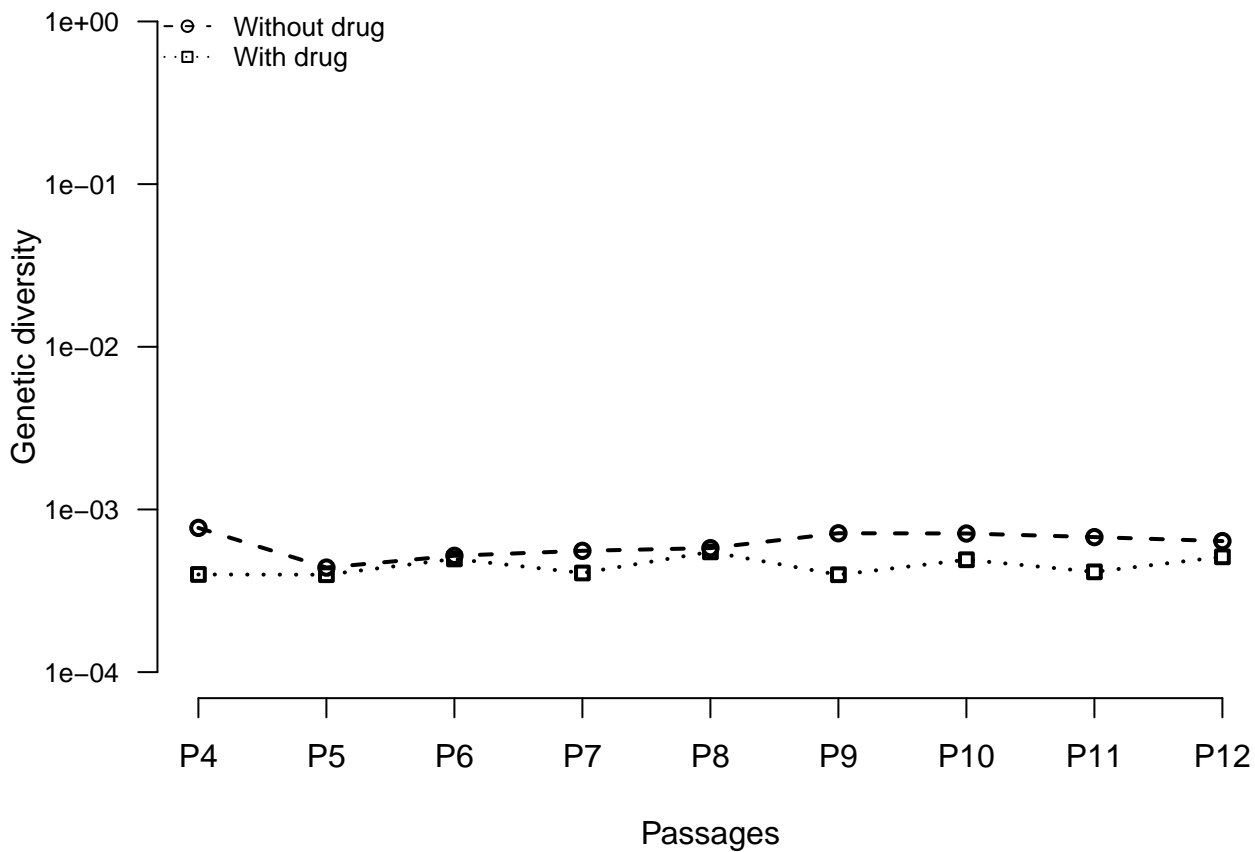

Supplement: Figure S1 — Genetic diversity of H1N1 throughout the experiment. The genetic diversity measured as the average expected heterozygosity in passages 4 to 12 of our experiment in the absence (dotted line) or presence (dashed line) of oseltamivir. (PDF) [file pgen.1004185.s001.pdf]

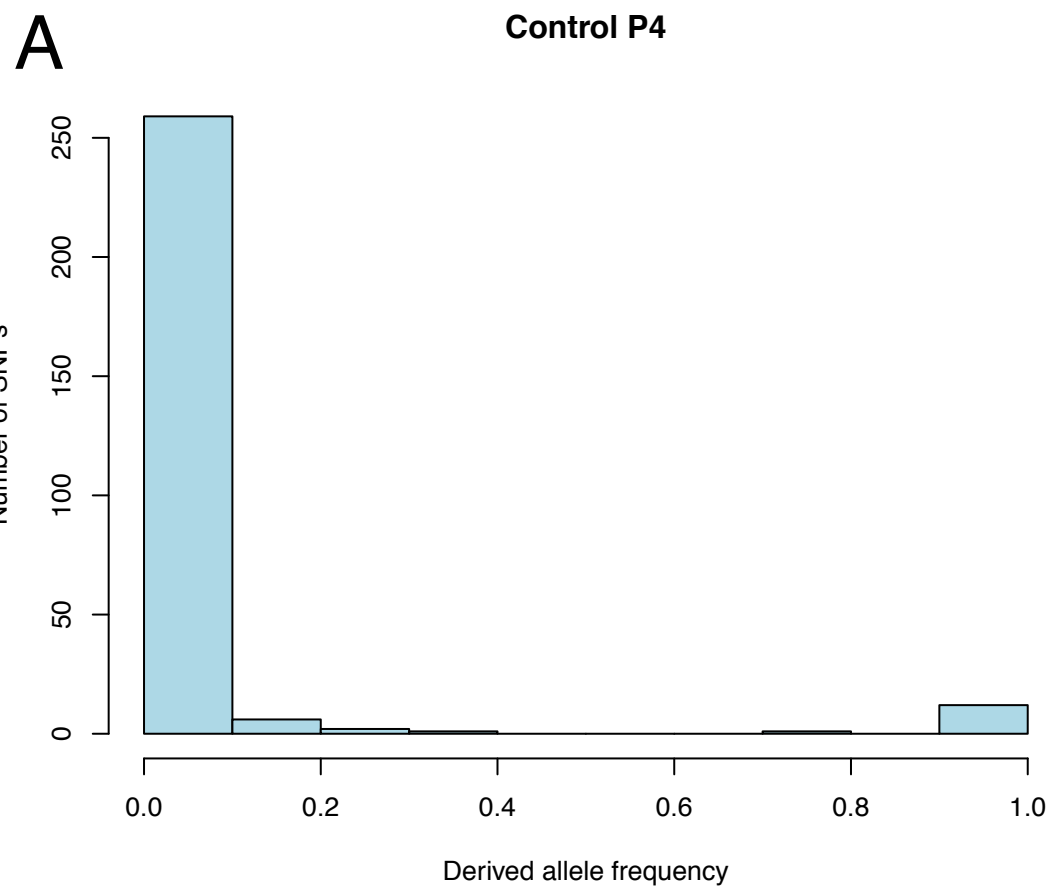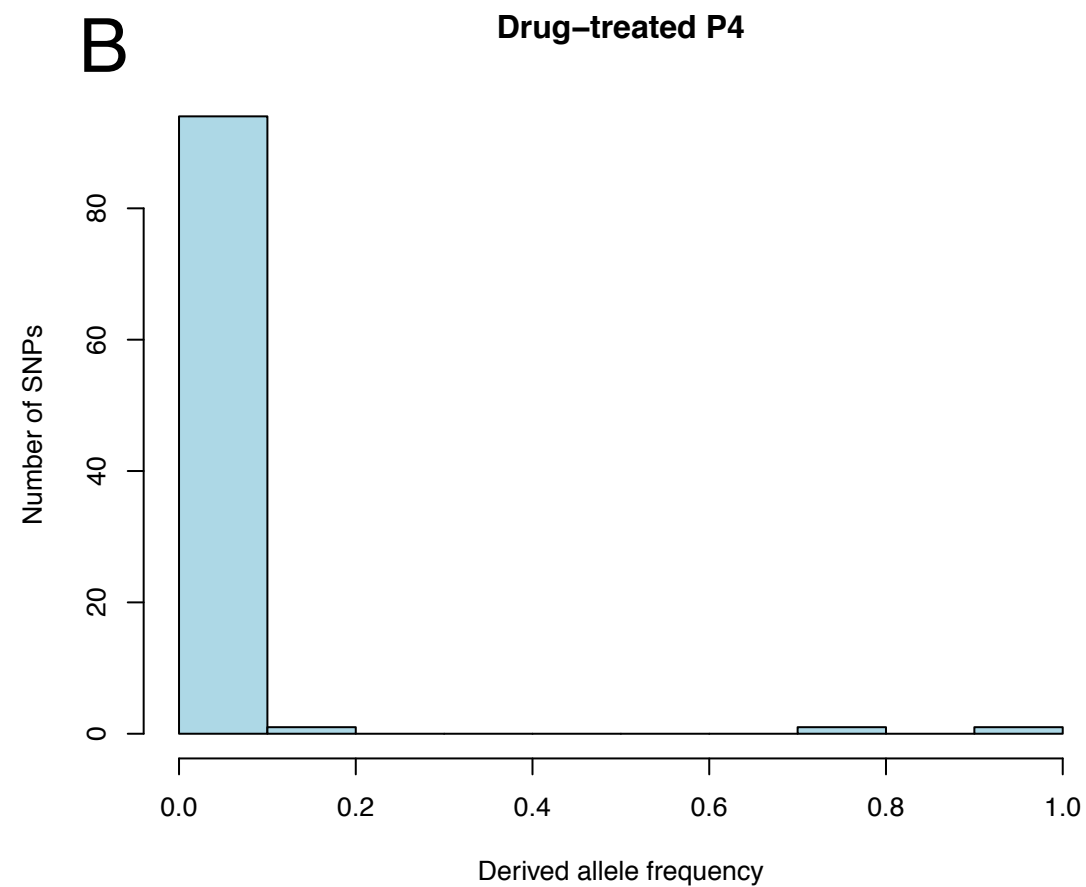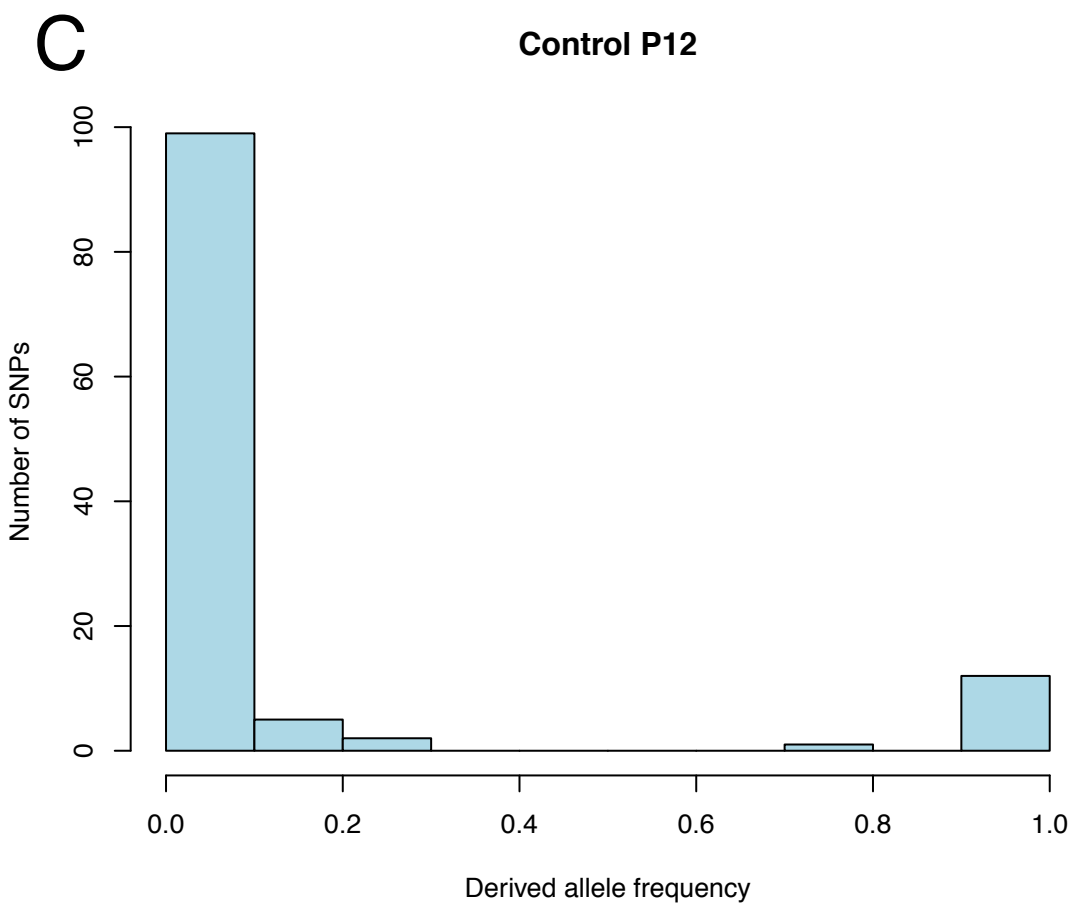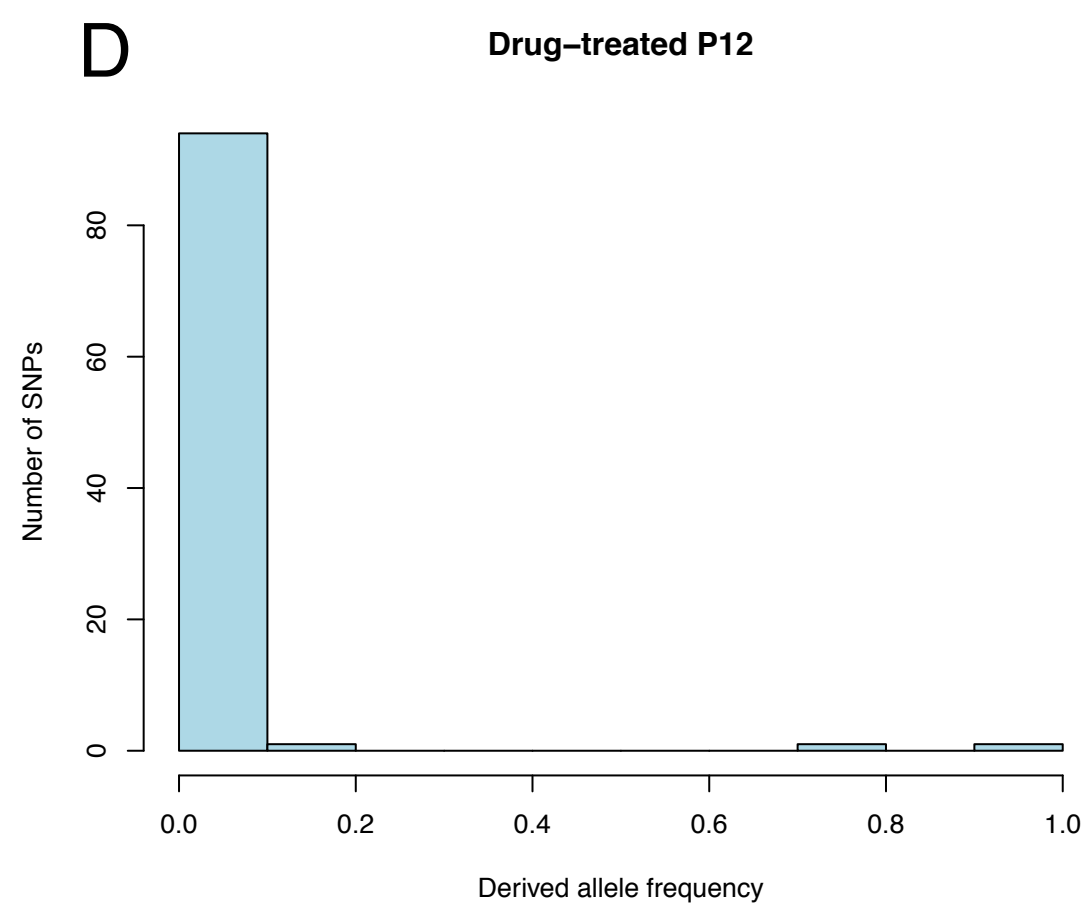

Supplement: Figure S2 — Site frequency spectra (SFS) of H1N1 populations during the experiment. The SFS at passages 4 (A and B) and 12 (C and D) is shown in the absence (A and C) and presence (C and D) of oseltamivir. (PDF) [file pgen.1004185.s002.pdf]

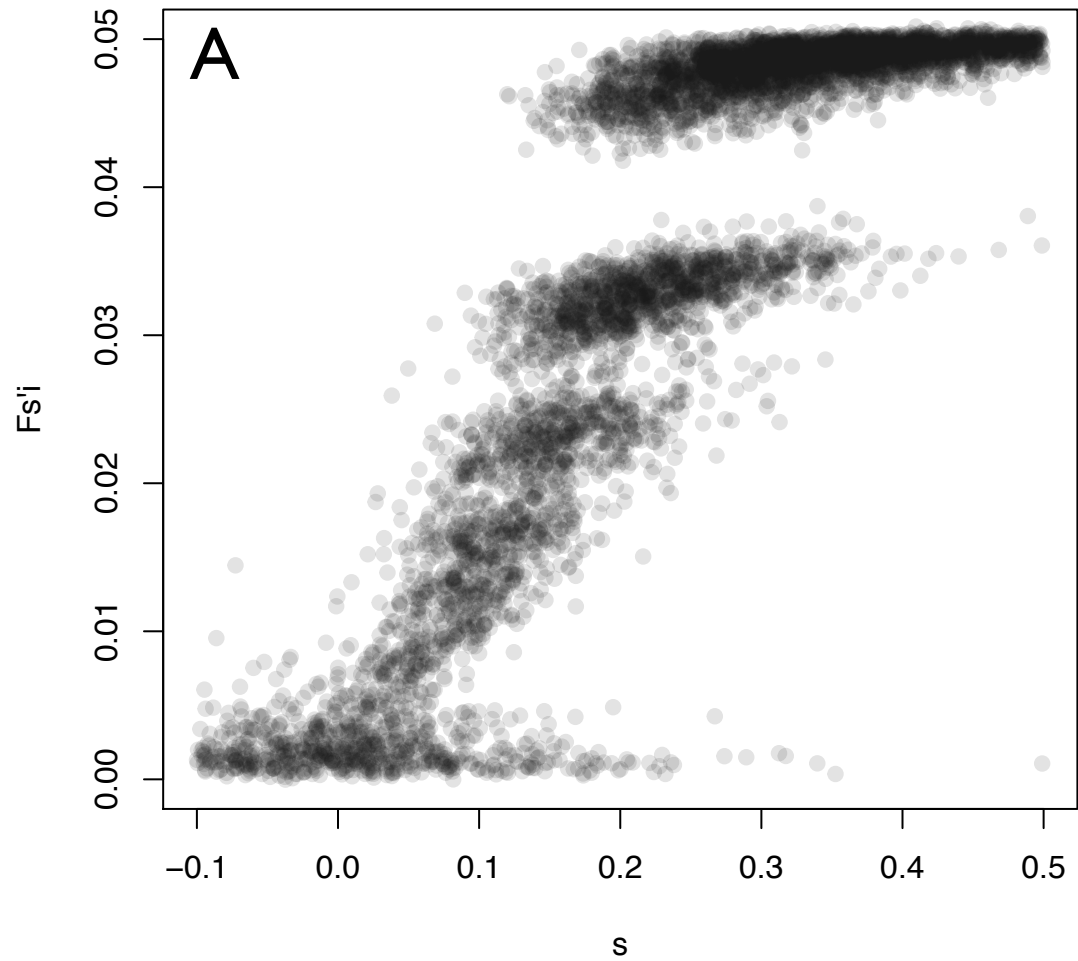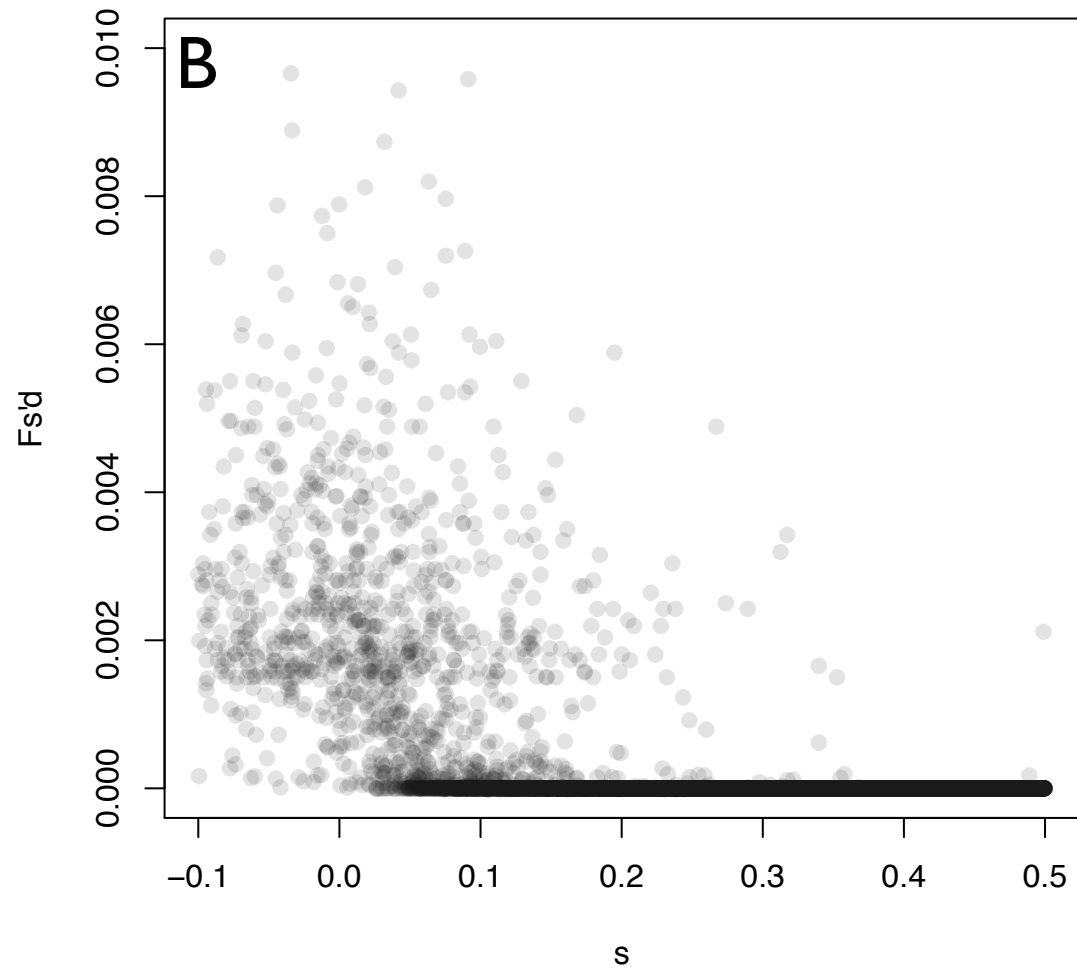

Supplement: Figure S3 — ABC correlation plot. The correlation between the simulated selection coefficients s and the two statistics (Fs′i (A) and Fs′d (B)) used in our ABC method. Note that Figure 4A is showing the same correlation using colors. (PDF) [file pgen.1004185.s003.pdf]

# Allele Frequency

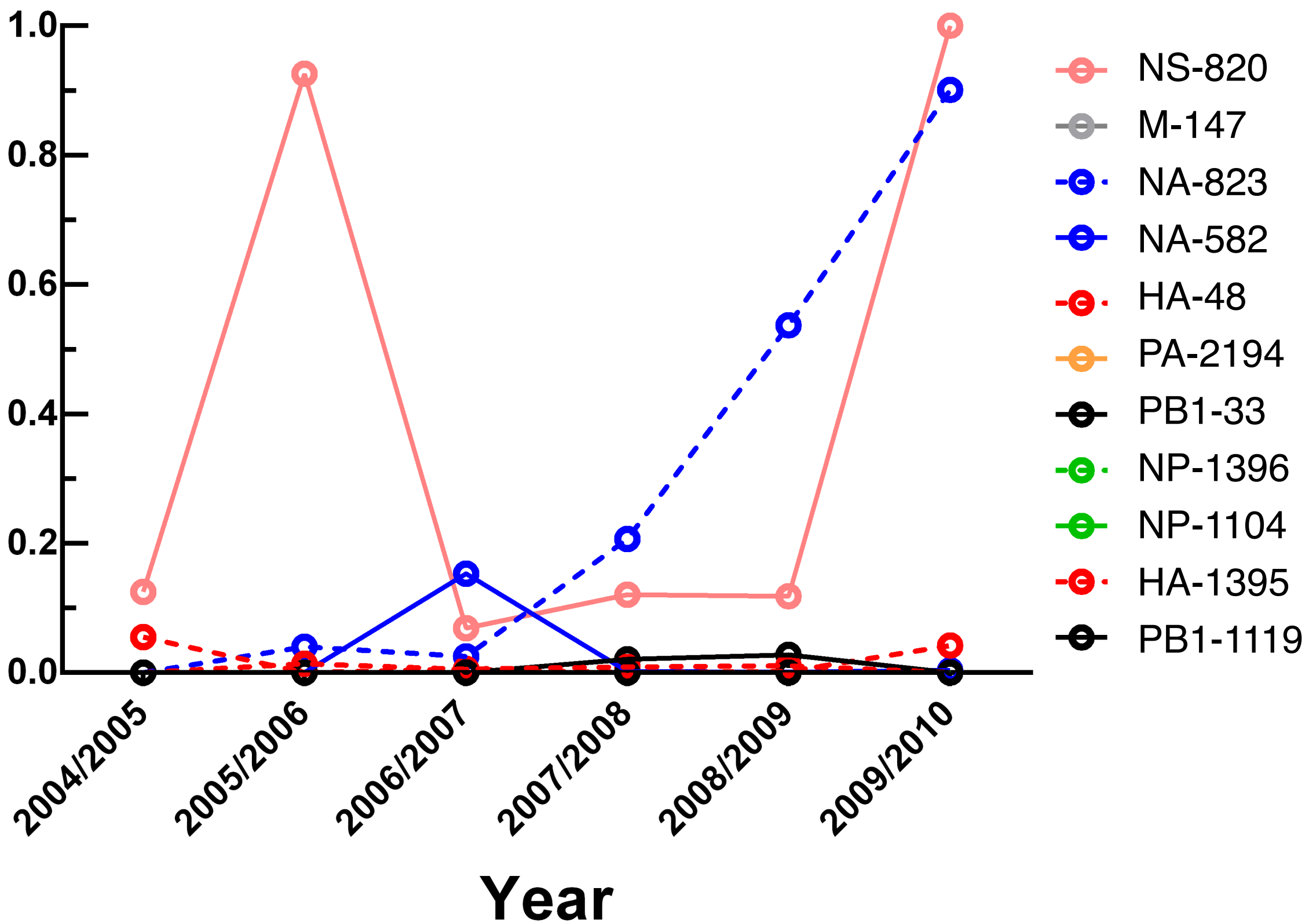

Supplement: Figure S4 — Frequency of identified beneficial mutations in natural populations. The allele frequency in natural populations from the NCBI Influenza Virus Resource database for the significant mutations identified to be under selection - plotted between years 2004 and 2010. (PDF) [file pgen.1004185.s004.pdf]

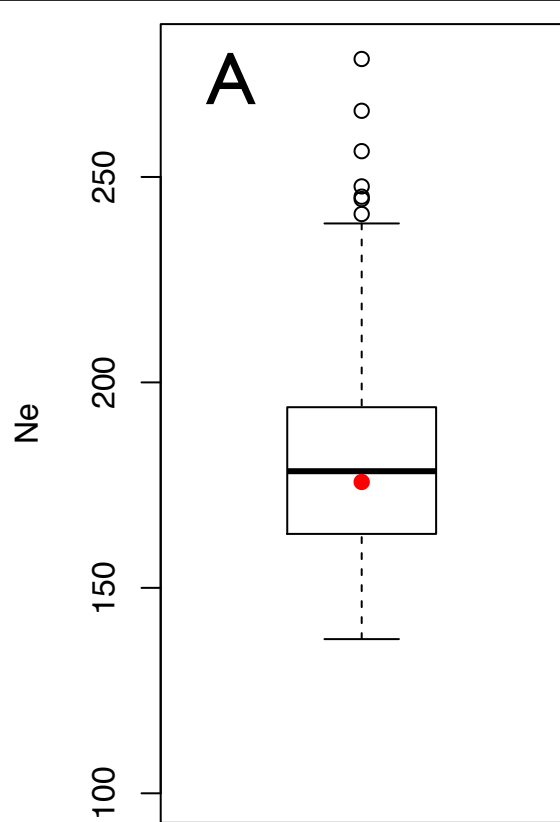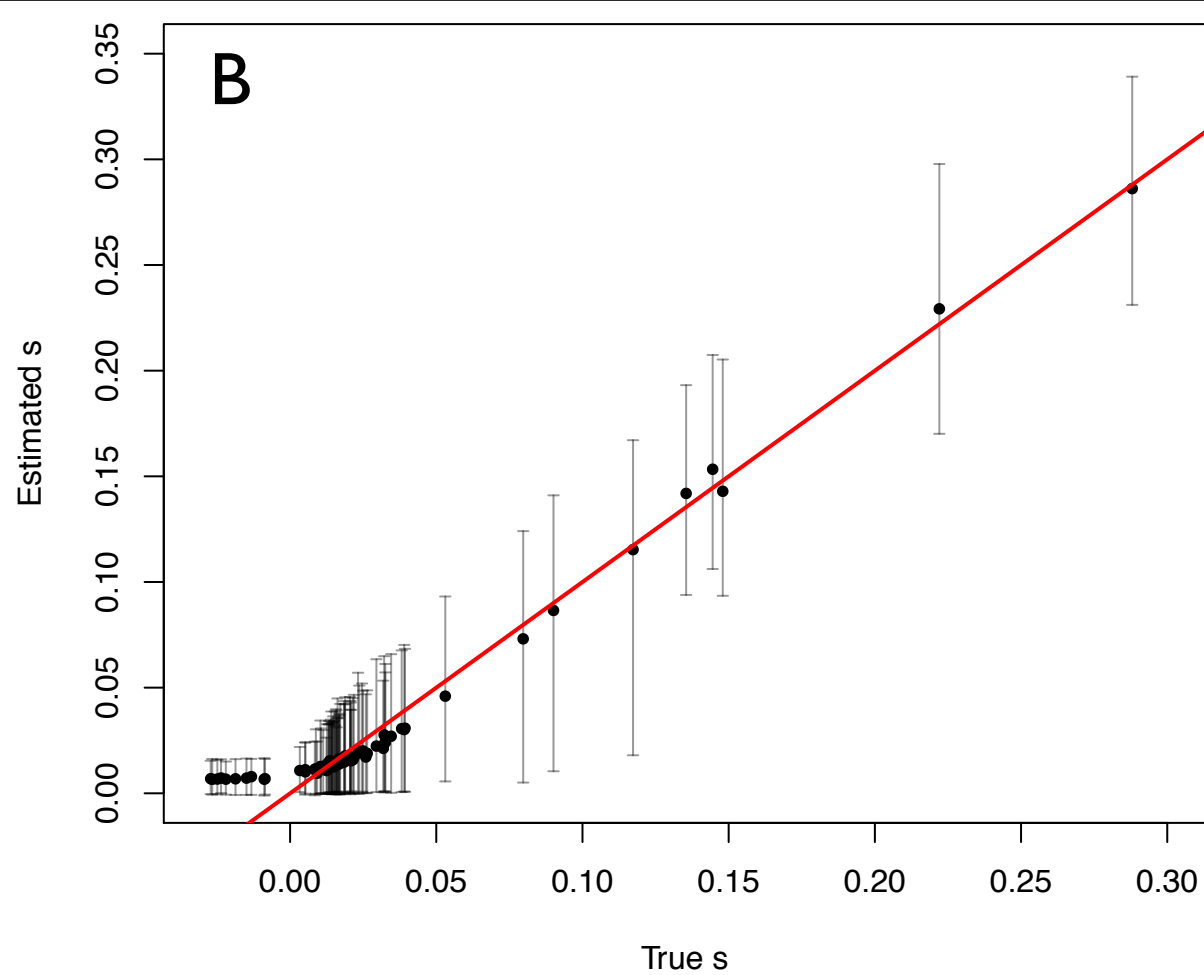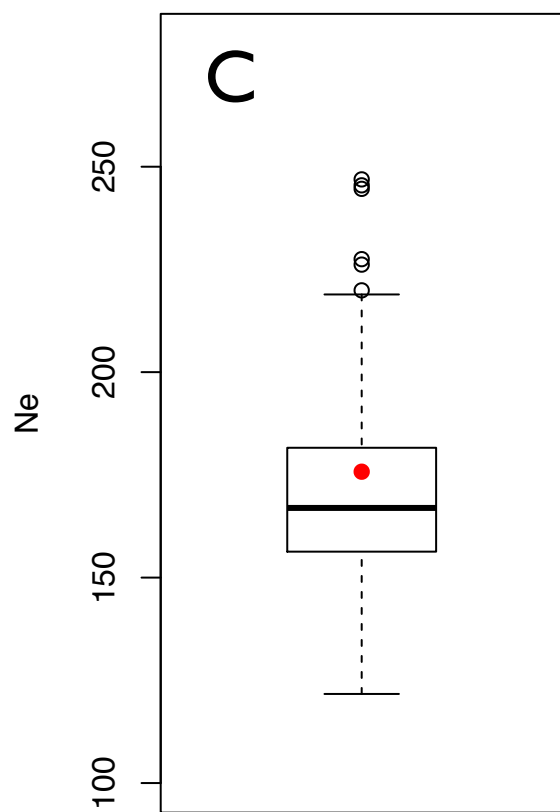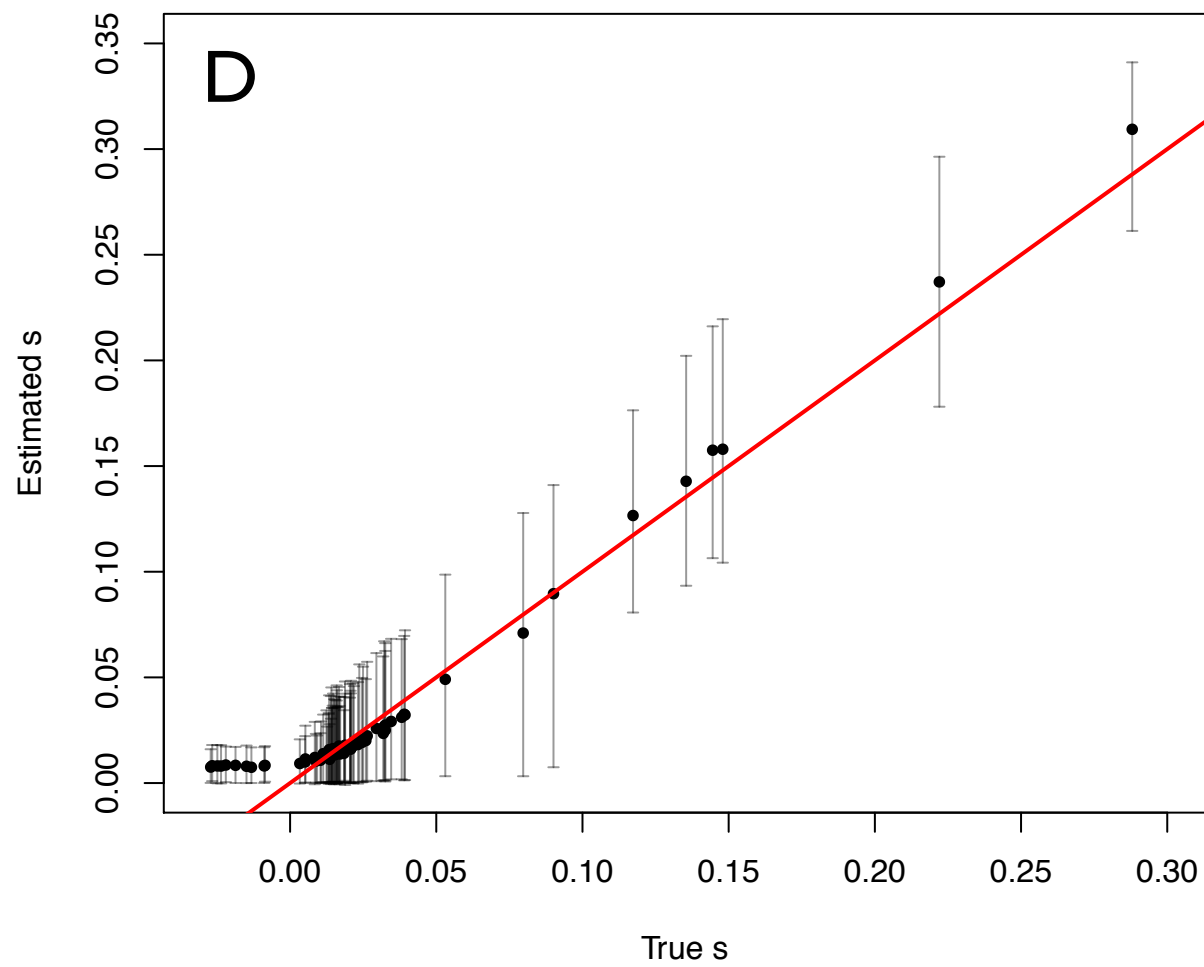

Supplement: Figure S5 — Cross validation of our Ne-based ABC method. The true vs. the estimated values of Ne (A and C) and s (B and D) for the 1000 simulated data used to validate our ABC procedure. We used similar parameters to our real data: sample size = 1000, Ne = 176 and initial allele frequencies of 1/Ne. We simulated a population of constant size (A and B) or experiencing recurrent bottleneck (N = 23) followed by exponential growth (up to N = 106) mimicking our experiment (C and D). Error bars in B and D represent the 10% and 90% quantiles over the 1000 replicates. The red dot in A and C and the red line in B and D indicate the true value. (PDF) [file pgen.1004185.s005.pdf]

**A**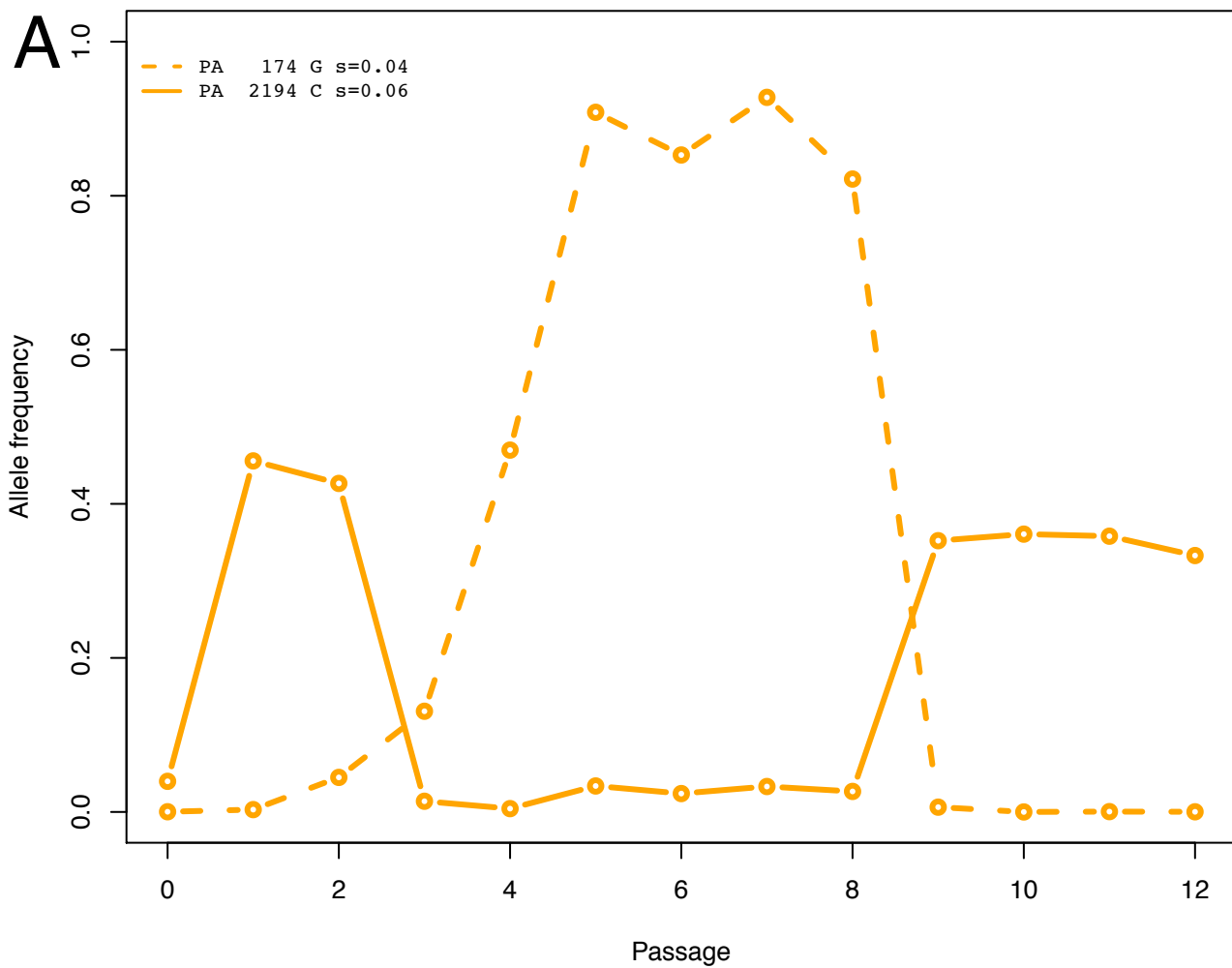**B**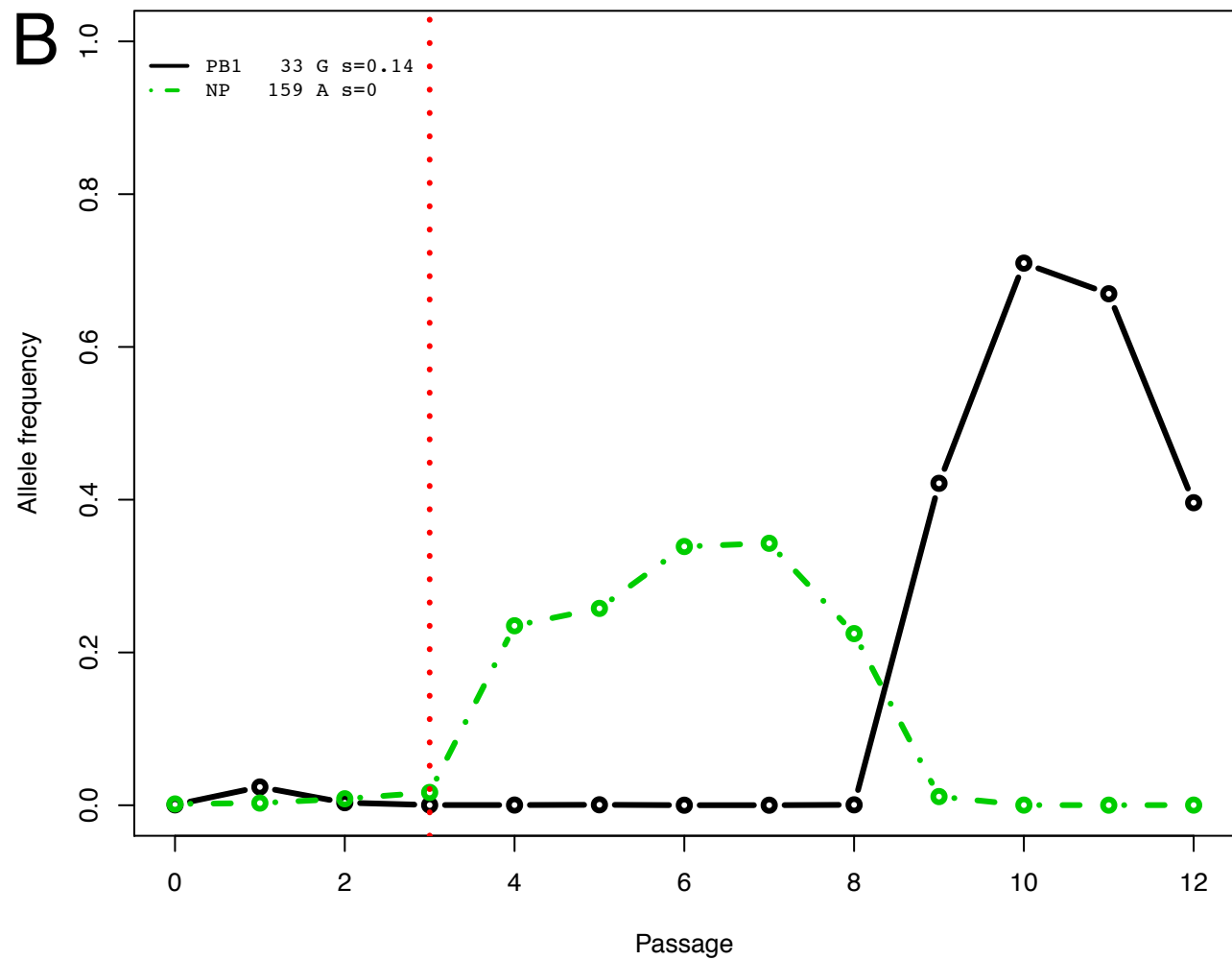

Supplement: Figure S6 — SNPs with poor fit to the Wright-Fisher model. The minor allele frequency trajectories of all SNPs identified as not fitting the Wright-Fisher model in the absence and presence of oseltamivir respectively in A and B. The horizontal dotted red line indicates the start of oseltamivir treatment (see Figure 1). Trajectories are represented in dashed lines if a second SNP was significant within the same segment. For each SNP, the name of the segment, the position of the SNP, the nucleotide increasing in frequency, and the estimated selection coefficients with our Ne-based ABC method are indicated in the top left corner of A and B. (PDF) [file pgen.1004185.s006.pdf]

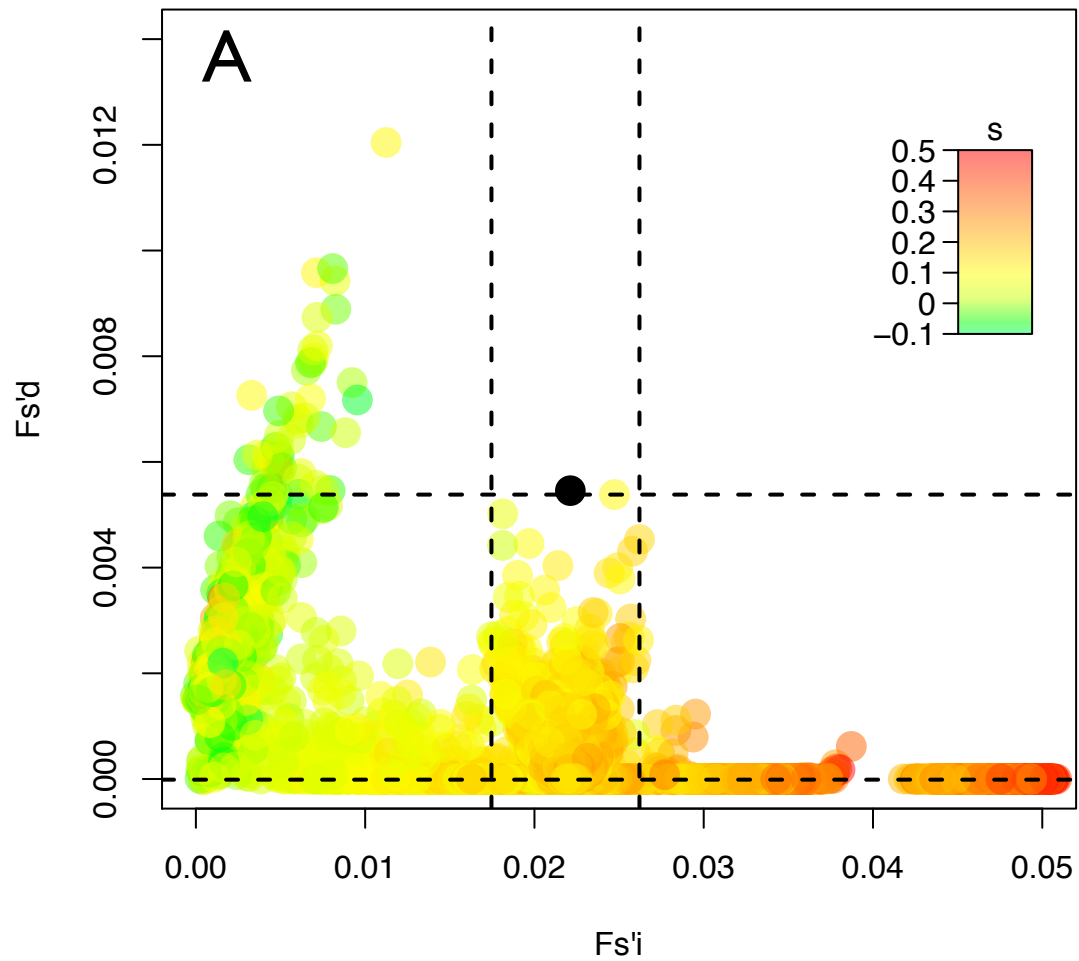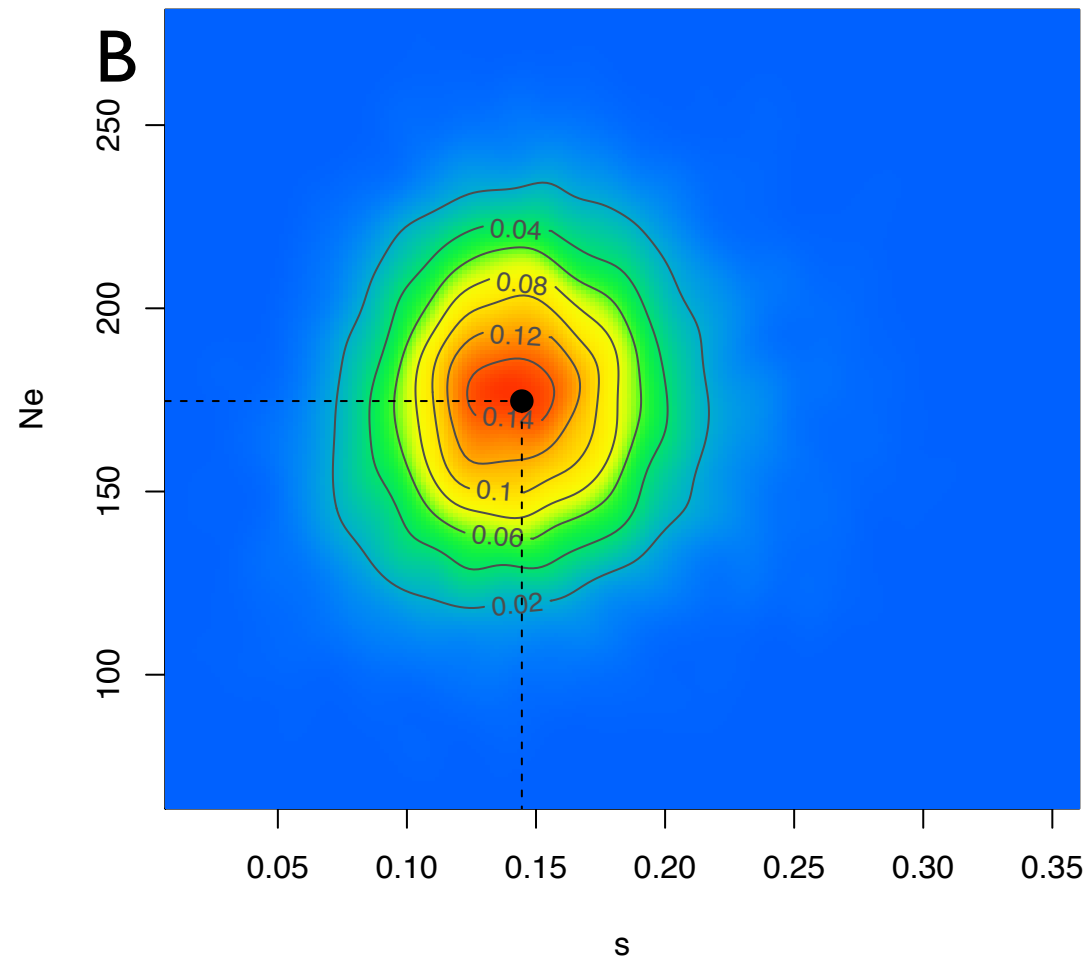

Supplement: Figure S7 — Ne-based Approximate Bayesian Computation for SNP PB1 33 (K11). For 10'000 simulated trajectories (out of the 100'000 simulations performed), we plot in A the values of the statistics Fs′i and Fs′d with colors corresponding to the selection coefficients s, as well as the values calculated for the real trajectory of the poor fitting PB1 33 (K11) mutation (see Figure S5) in black. We indicate the region corresponding to the best 1% retained simulations with a dashed line, and we plot the corresponding two-dimensional posterior distribution for s and Ne in B. We clearly see in A the inability of the model to generate simulations near the observed data, with the black dot being outside the retained regions defined by the dashed lines. (PDF) [file pgen.1004185.s007.pdf]

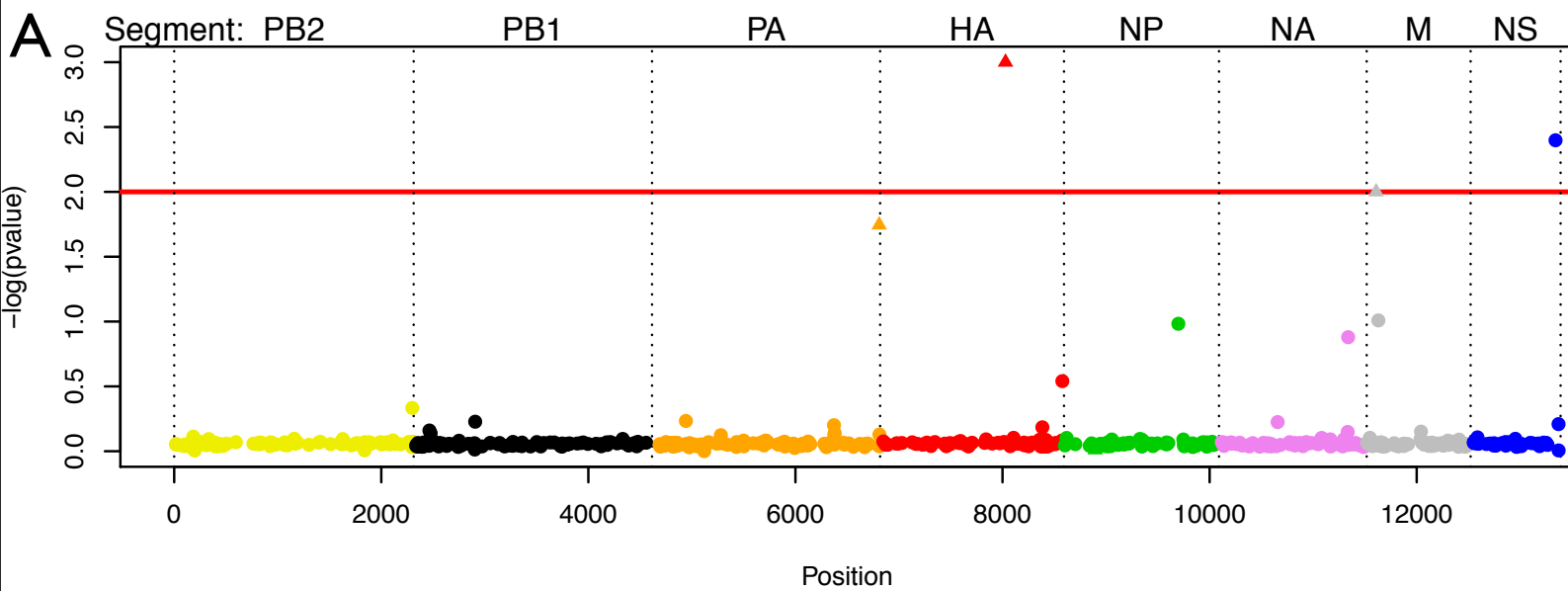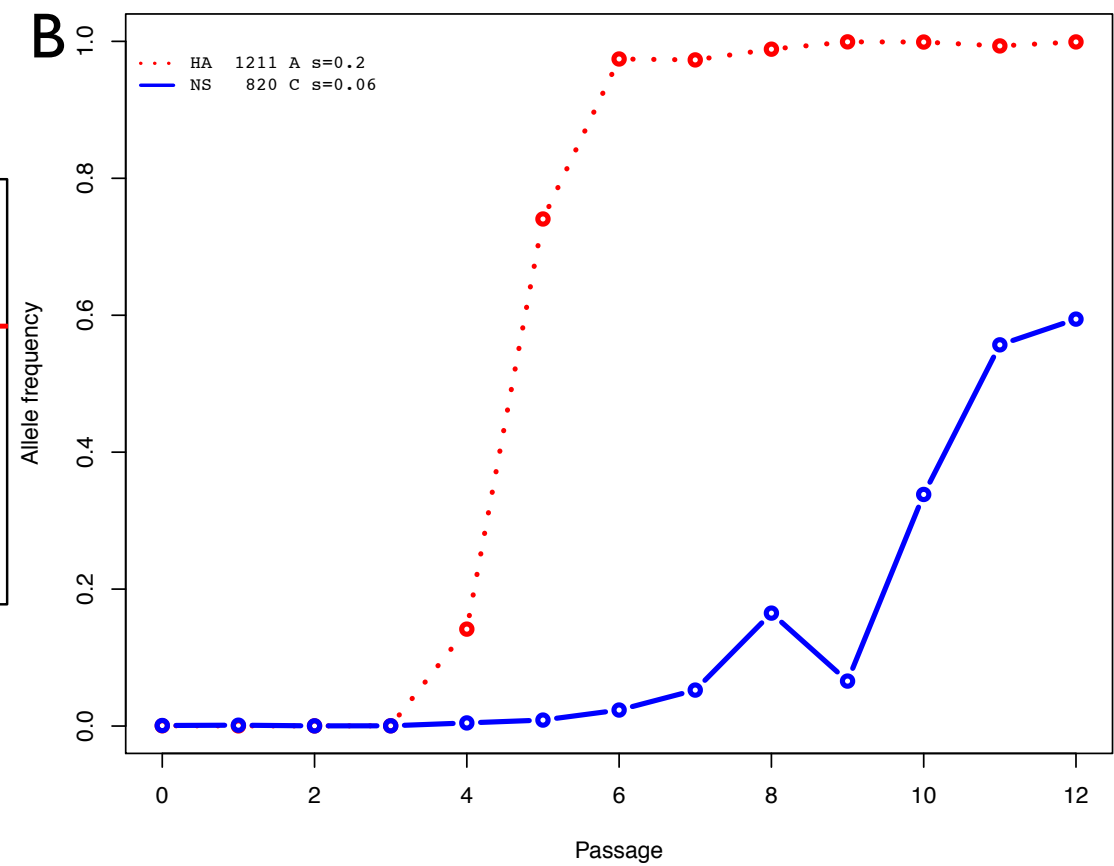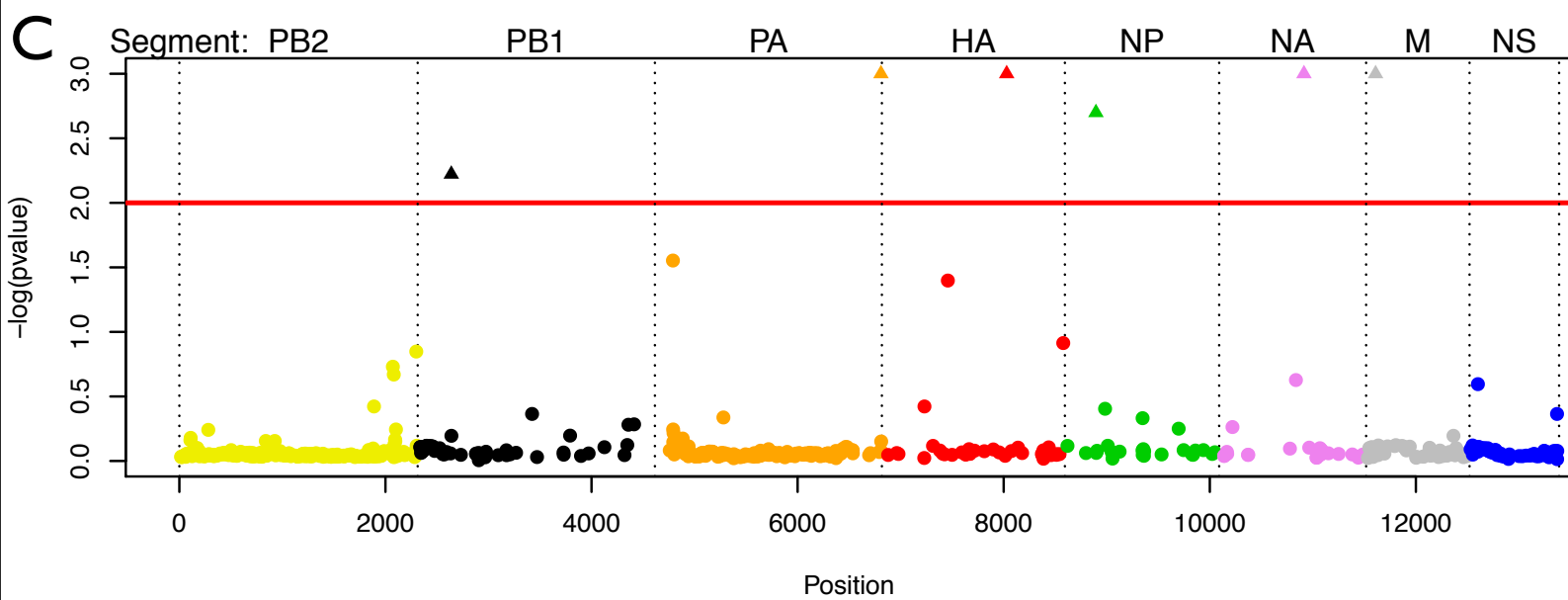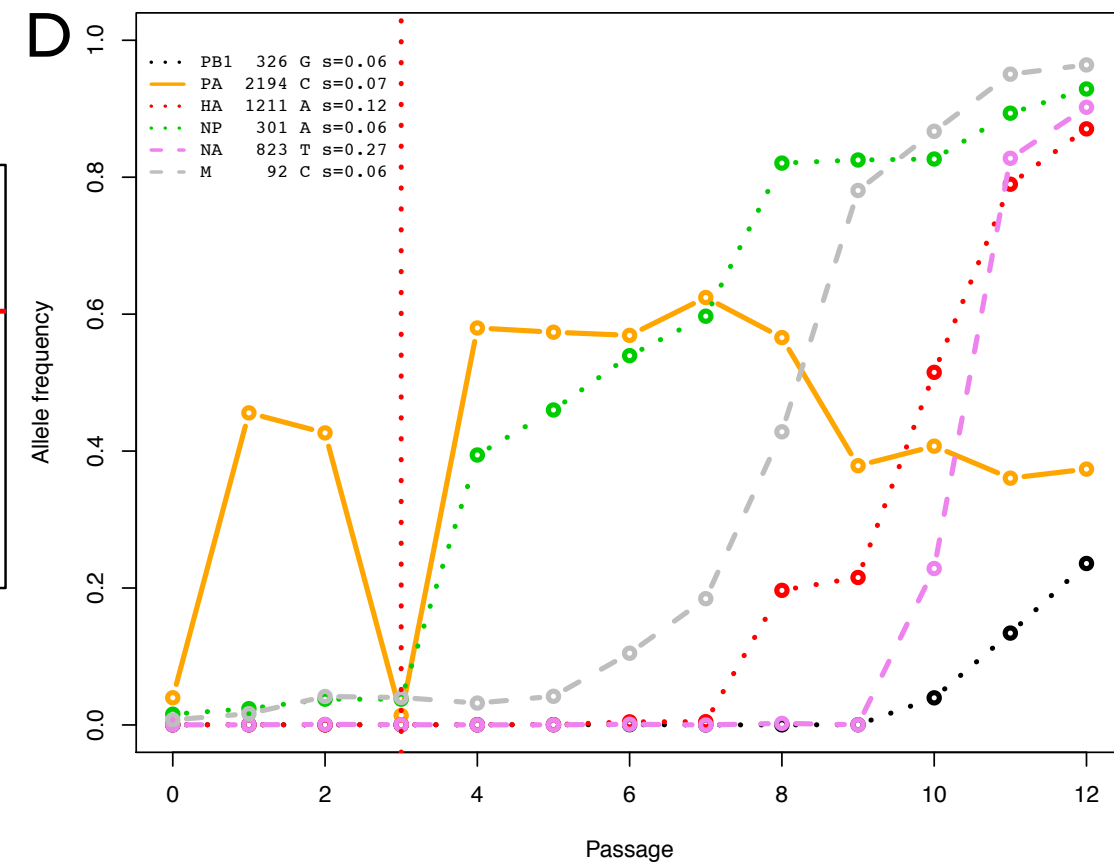

Supplement: Figure S8 — Evidence of positive selection in the H1N1 genome in the absence and presence of oseltamivir for replicated data. We plot the Bayesian P-values of each SNP in log scale in the absence and presence of oseltamivir in A and C, respectively. The horizontal red lines are genome-wide significance thresholds of P = 0.01. The eight segments are separately color-coded, a scheme which is maintained in all panels and in Figure 3. Significant nonsynonymous mutations are represented with triangles. We plot the minor allele frequency trajectories of all significant SNPs over the replicated experiment in the absence and presence of oseltamivir respectively in B and D. The horizontal dotted red line indicates the start of oseltamivir treatment (see Figure 1). All colors and line styles match those in Figure 1. Trajectories are represented as dashed lines when a second SNP was significant in a segment, and dotted lines for a third SNP. For each significant SNP, the name of the segment, the position of the SNP, the nucleotide increasing in frequency, and the estimated selection coefficients with our Ne-based ABC method are indicated in the top left corner of B and D. (PDF) [file pgen.1004185.s008.pdf]

Log likelihood

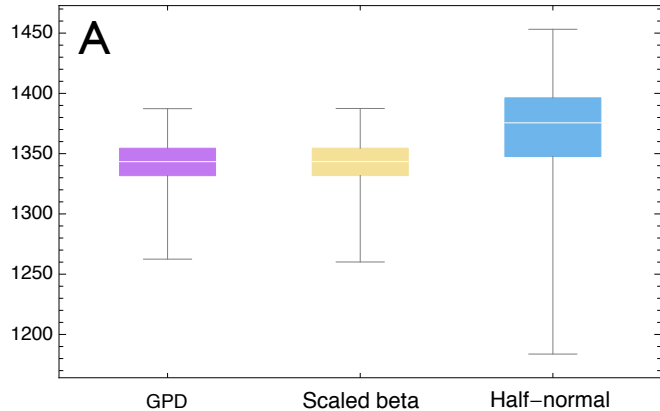

Log likelihood

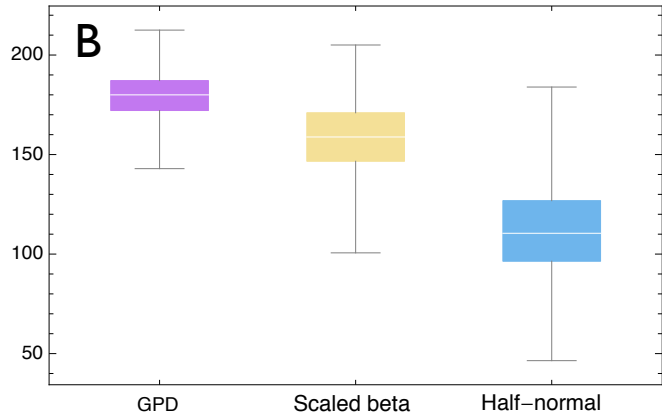

Supplement: Figure S9 — Maximum likelihood for the DFE fit. The boxplots show the distribution of the maximum log-likelihoods obtained from 1000 samples of a weighted likelihood bootstrap in the absence and presence of oseltamivir in A and B, respectively. (PDF) [file pgen.1004185.s009.pdf]

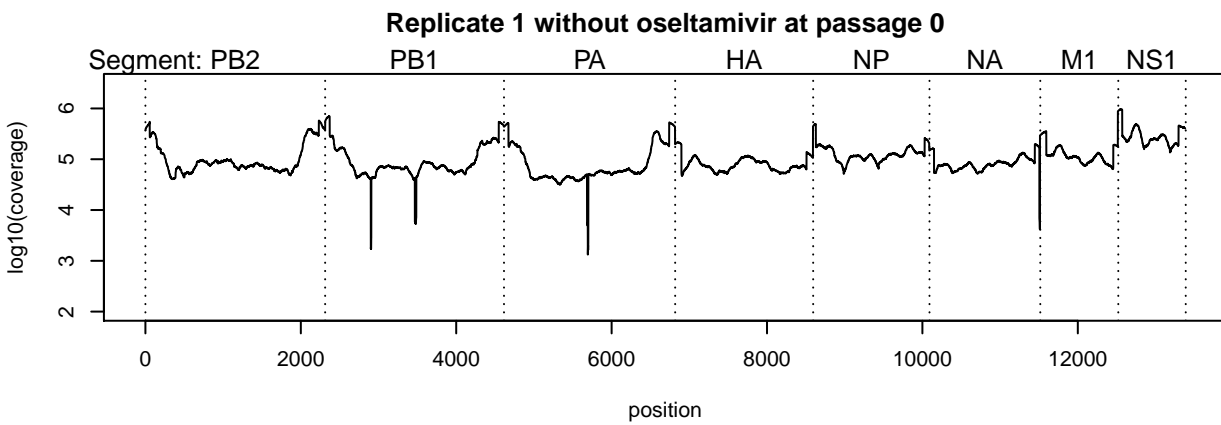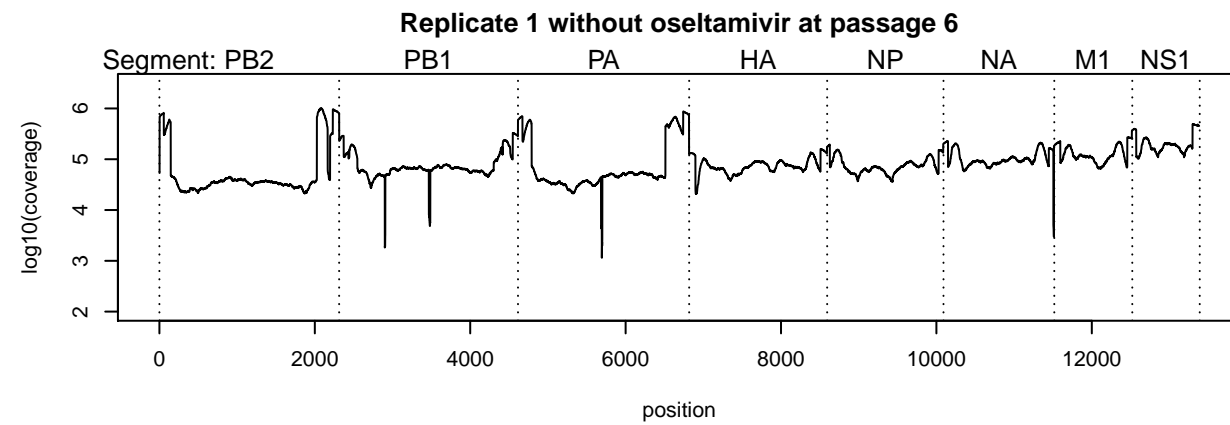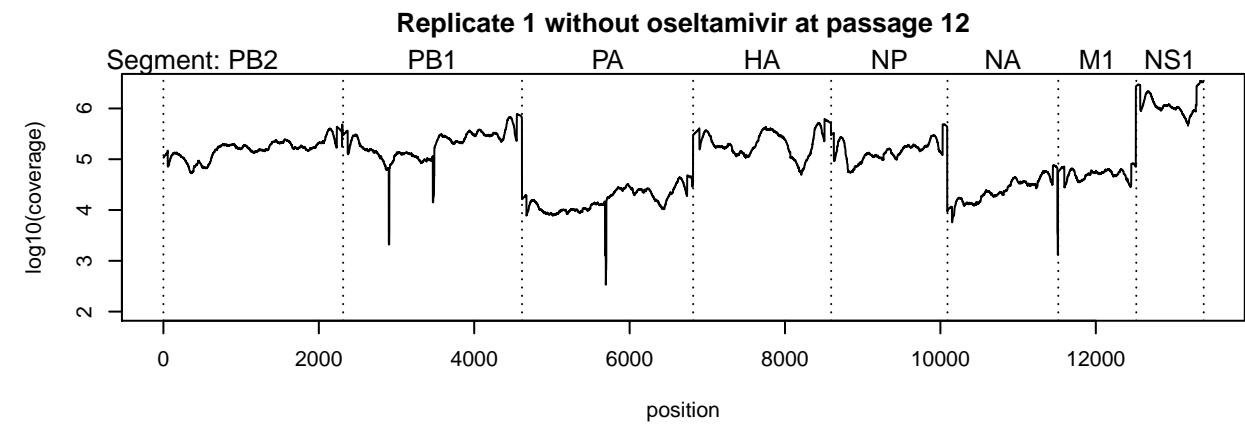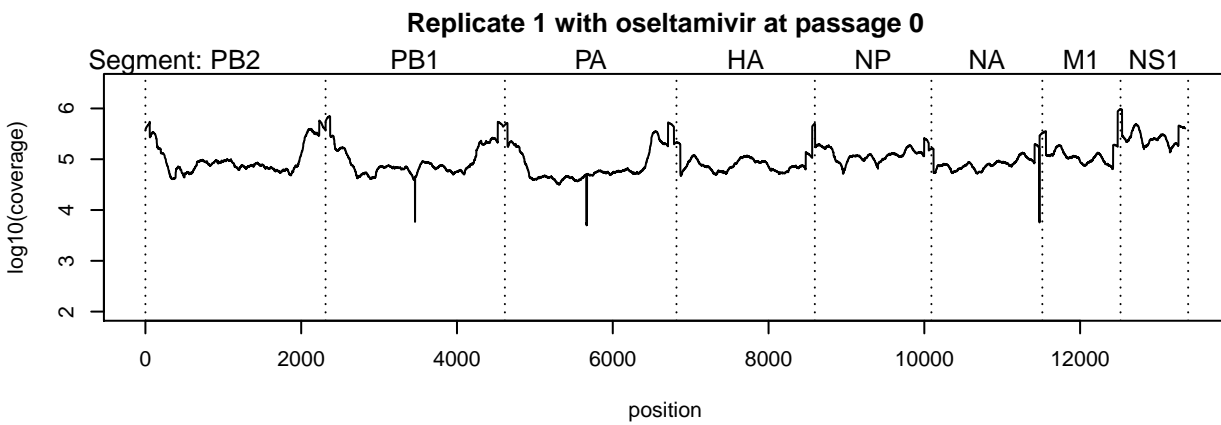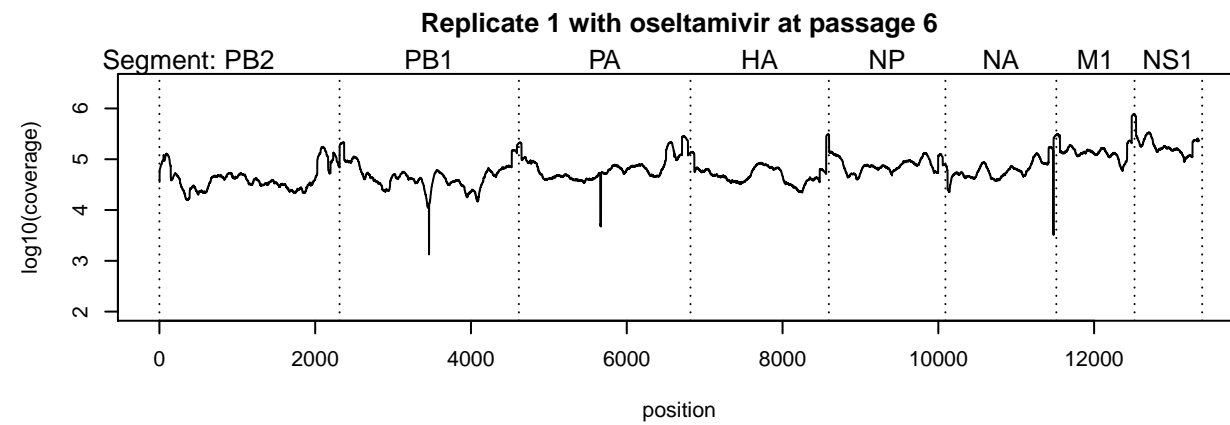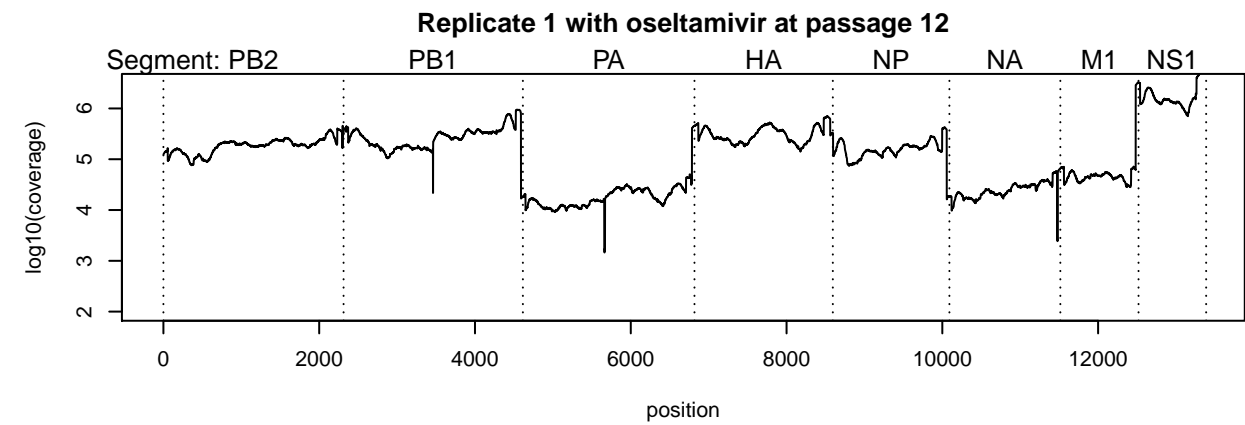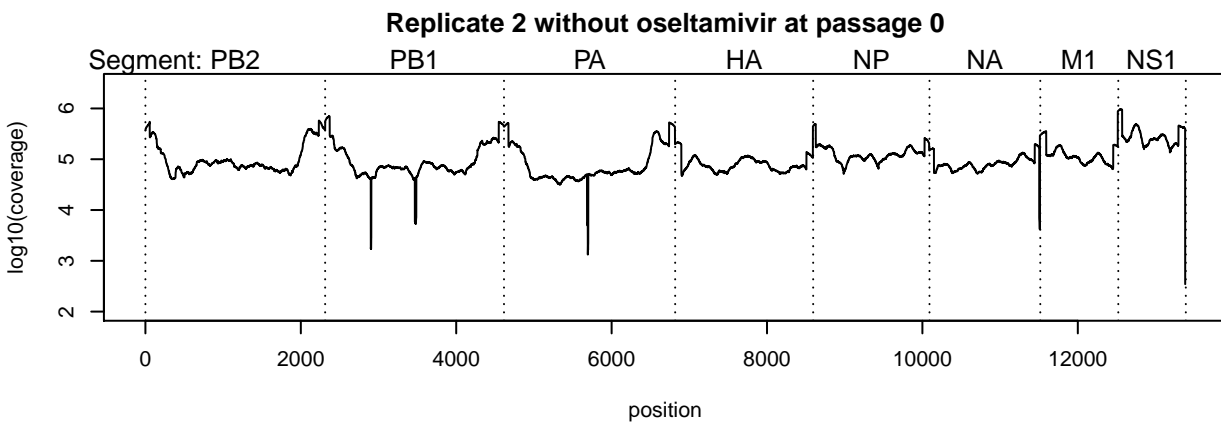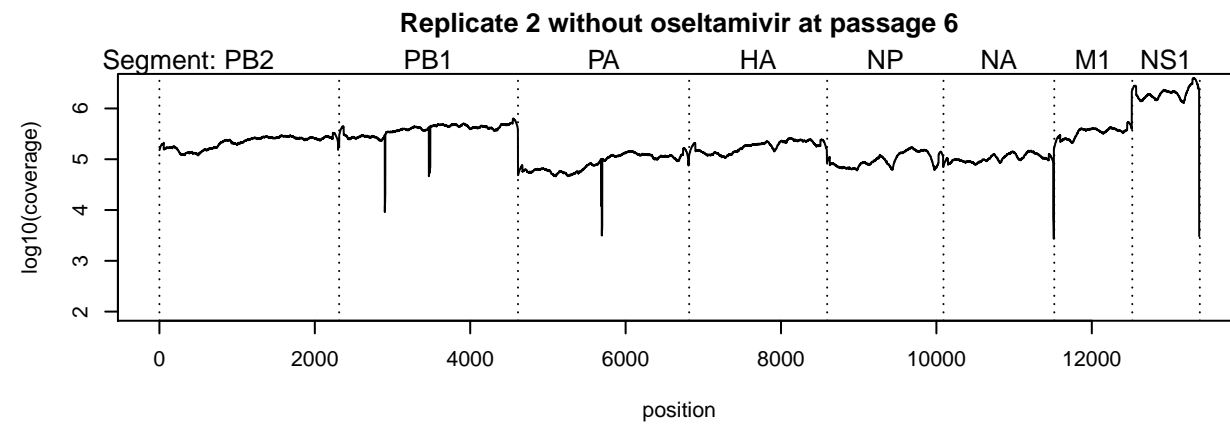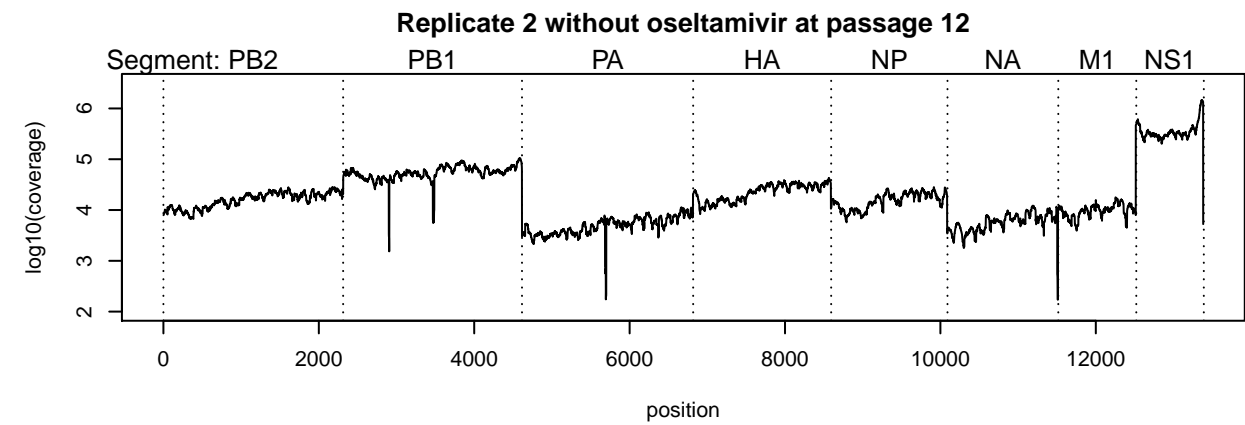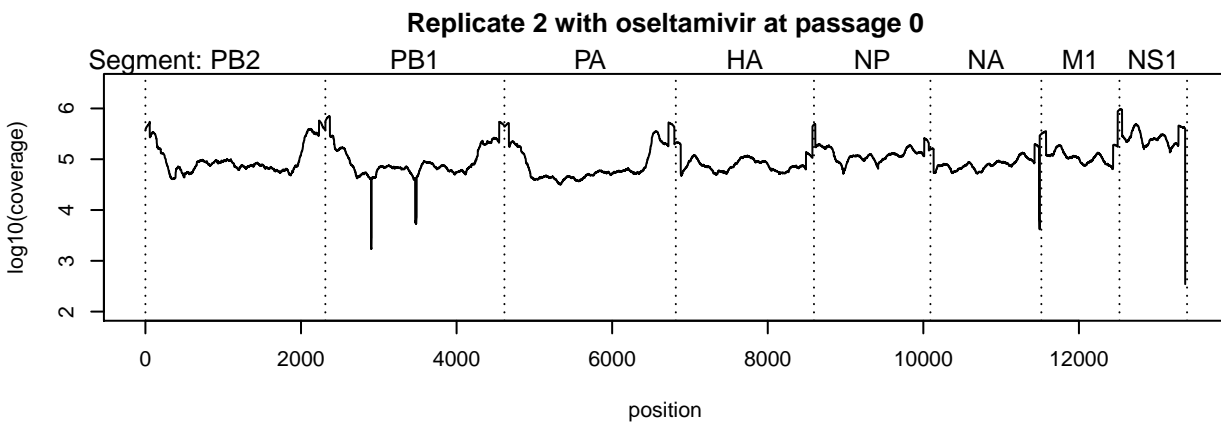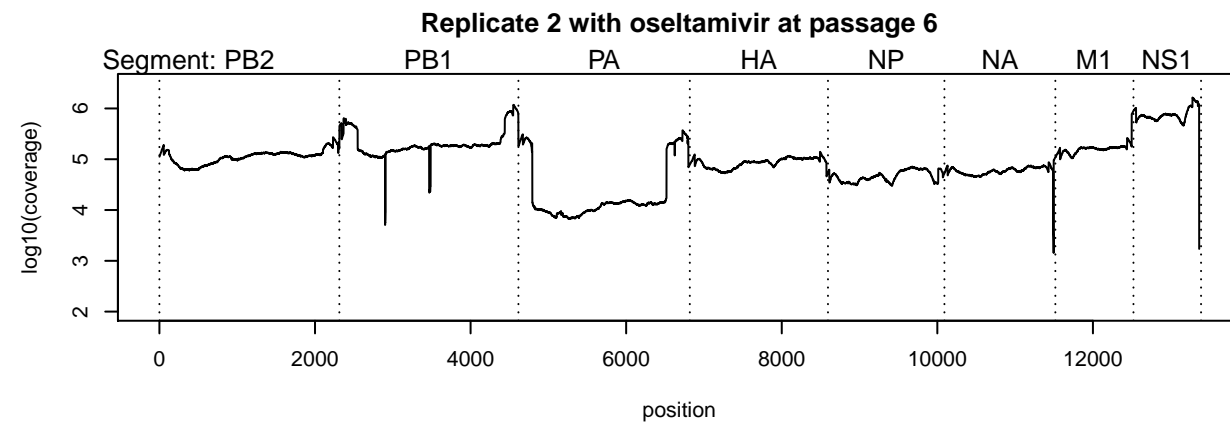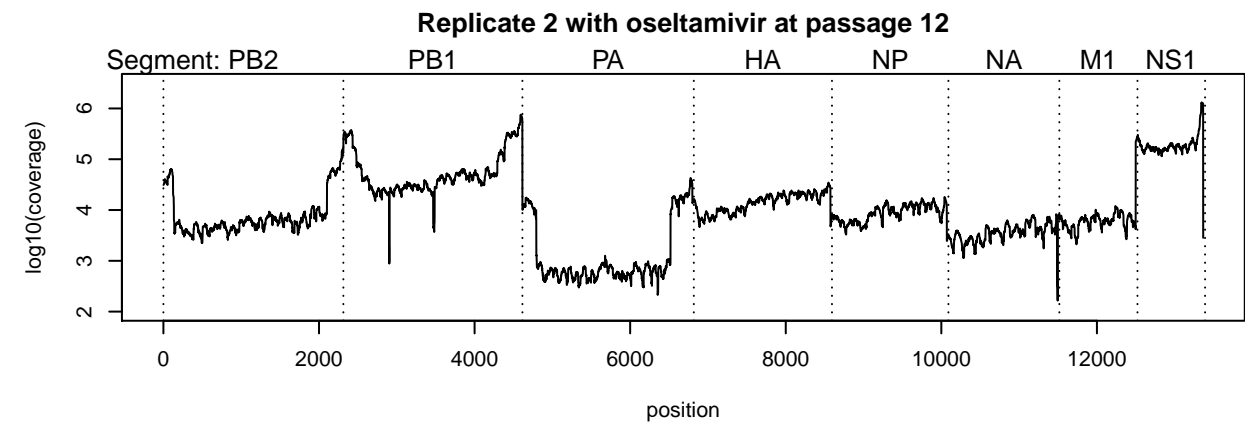

Supplement: Figure S10 — Genome wide sequence coverage data for samples used in this study. The coverage in log scale for our four experiments at passages 0, 6 and 12 (see Figure 1). (PDF) [file pgen.1004185.s010.pdf]

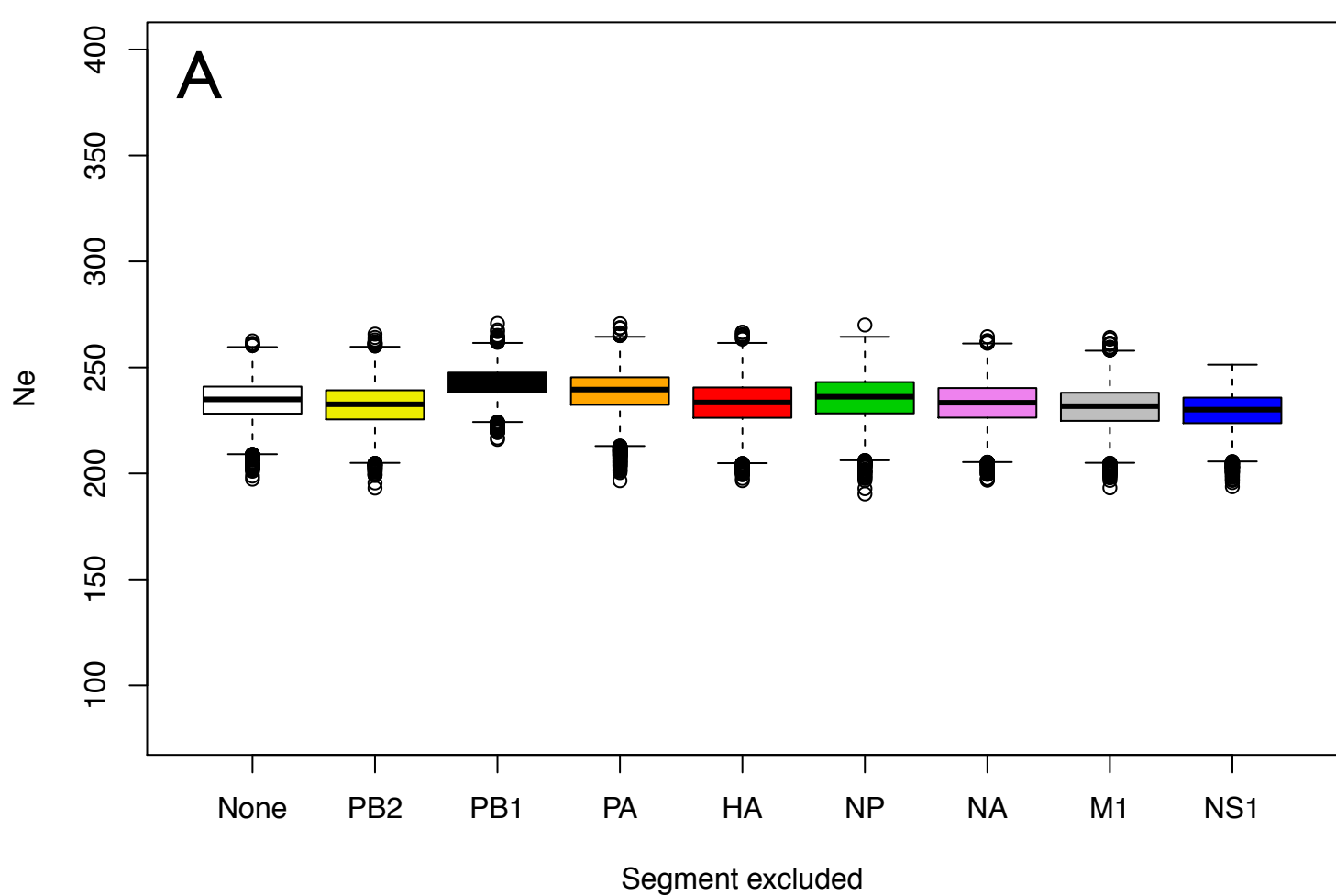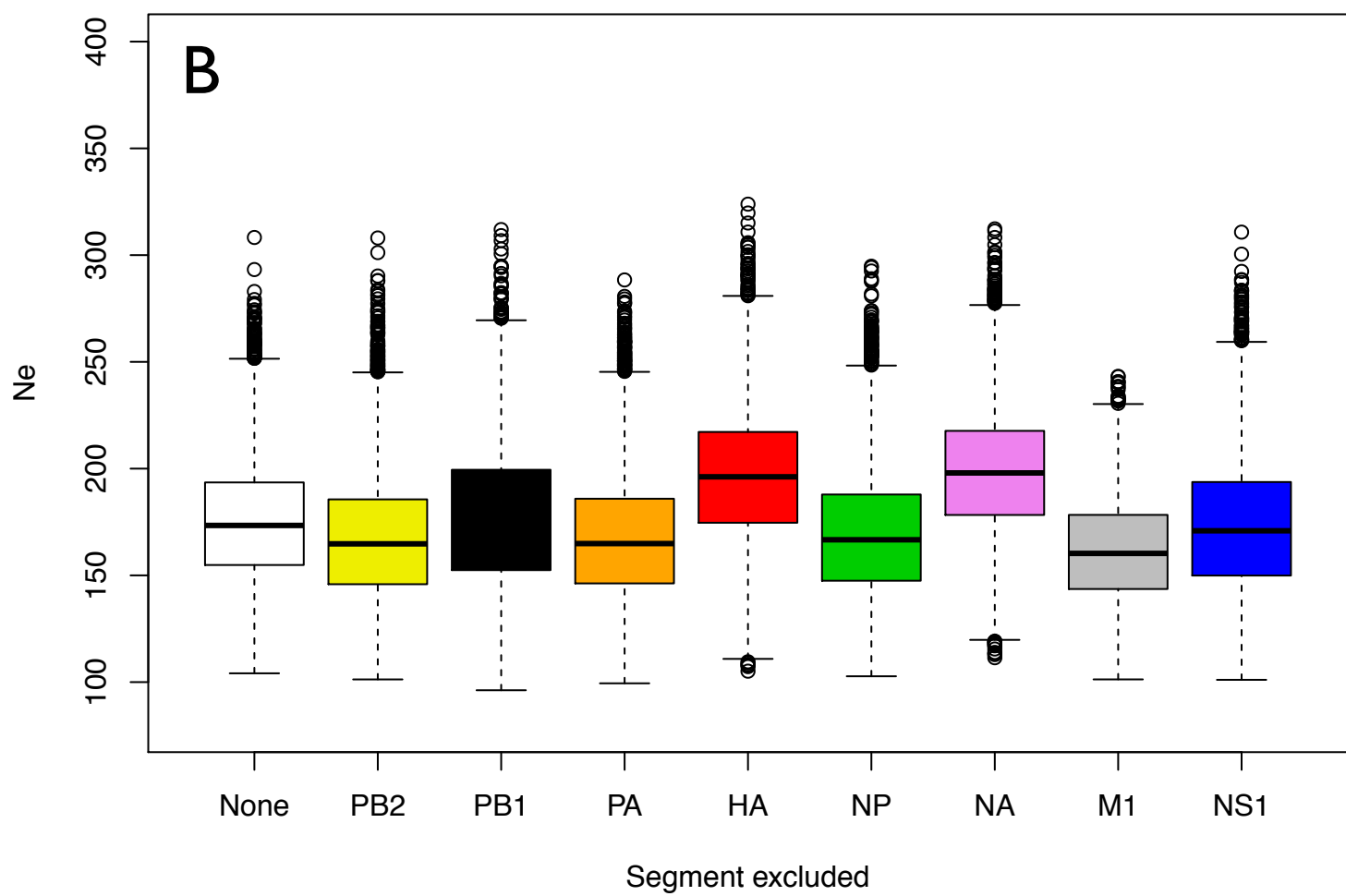

Supplement: Figure S11 — Estimated Ne when excluding segments. The posterior distribution obtained for Ne using step 1 of our ABC algorithm when excluding each segment one by one in the absence (A) and presence (B) of oseltamivir. Segment colors match those in Figure 3 and S8. (PDF) [file pgen.1004185.s011.pdf]
