# Supplementary material for: Influenza Virus Drug Resistance: A Time-Sampled Population Genetics Perspective
Source: PLoS Genet. 2014 Feb 27;10(2):e1004185. doi: 10.1371/journal.pgen.1004185 (PMC3937227; doi:10.1371/journal.pgen.1004185)
Supplement: Table S1 — Estimated selection coefficients for the replicate experiment. Comparison of Ne-ABC and Malaspinas et al. [32] estimates of s for the significant trajectories under selection for the replicate experiment. Bold indicates nonsynonymous mutations. We indicate the nucleotide corresponding to the minor allele, with its initial frequency at the beginning of the experiment in the absence of oseltamivir, or at passage 4 when drug treatment began (see Figure 1). For the Ne-ABC method, we give the 99% highest posterior density intervals (HPDIs) in brackets. (PDF) [file pgen.1004185.s012.pdf]

	Segment	Position	Protein	Sequential numbering	Other numbering	Allele	Initial frequency	Final frequency	*Ne*-ABC *s* estimates (99% HPDIs)	Malaspinas *et al. [32]* *s* estimates
Without oseltamivir	**HA**	**1211**	**HA2**	**N393K**	**N50K (HA2)**	**A**	**0.04%**	**100.0%**	**0.20 (0.08;0.35)**	**0.28**
	NS	820	NS2	F116		C	0.08%	59.5%	0.06 (0.01;0.12)	0.09
With oseltamivir	**PB1**	**326**	**PB1**	**E109G**		**G**	**0.02%**	**27.3%**	**0.06 (0.01;0.12)**	**0.08**
	**PA**	**2194**	**PA**	**Noncoding**		**C**	**1.4%**	**37.4%**	**0.07 (0.01;0.13)**	**0.00**
	**HA**	**1211**	**HA2**	**N393K**	**N50K (HA2)**	**A**	**0.04%**	**87.6%**	**0.12 (0.05;0.20)**	**0.08**
	**NP**	**301**	**NP**	**D101N**		**A**	**3.8%**	**92.9%**	**0.06 (0.01;0.12)**	**0.05**
	**NA**	**823**	**NA**	**H275Y**	**H274Y (N2)**	**T**	**0.04%**	**90.3%**	**0.27 (0.12;0.48)**	**0.22**
	**M**	**92**	**M1**	**E23Q**		**C**	**4.0%**	**96.8%**	**0.06 (0.01;0.12)**	**0.11**
