# Supplementary material for: Influenza Virus Drug Resistance: A Time-Sampled Population Genetics Perspective
Source: PLoS Genet. 2014 Feb 27;10(2):e1004185. doi: 10.1371/journal.pgen.1004185 (PMC3937227; doi:10.1371/journal.pgen.1004185)
Supplement: Table S2 — Viral passaging and drug concentration data for samples used in this study. (PDF) [file pgen.1004185.s013.pdf]

		Without Oseltamivir	With Oseltamivir	
	**Passage**	**MOI**	**MOI**	**Drug Concentration (uM)**
**Replicate 1**	1	1.9E-04	1.9E-04	0
	2	1.7E-04	1.7E-04	0
	3	1.0E-02	1.0E-02	0
	4	1.0E-02	1.0E-02	0.1
	5	3.4E-01	2.0E-01	0.4
	6	3.4E-02	3.4E-02	0.8
	7	1.1E-01	2.9E-02	0.88
	8	1.9E-02	1.9E-02	1.2
	9	1.5E-04	7.4E-05	1.6
	10	3.7E-04	3.0E-04	3.2
	11	8.4E-03	8.3E-03	6.4
	12	1.2E-04	1.7E-04	12.8
**Replicate 2**	1	1.9E-04	1.9E-04	0
	2	1.7E-04	1.7E-04	0
	3	1.0E-02	1.0E-02	0
	4	1.0E-03	1.0E-03	0.1
	5	2.8E-04	5.0E-03	0.4
	6	1.1E-04	3.2E-02	0.8
	7	1.7E-04	3.2E-02	0.88
	8	2.2E-04	2.8E-03	1.2
	9	1.2E-05	1.4E-04	1.6
	10	4.2E-06	5.7E-04	3.2
	11	3.6E-04	9.1E-04	6.4
	12	2.1E-05	5.3E-03	12.8
