# Supplementary material for: Influenza Virus Drug Resistance: A Time-Sampled Population Genetics Perspective
Source: PLoS Genet. 2014 Feb 27;10(2):e1004185. doi: 10.1371/journal.pgen.1004185 (PMC3937227; doi:10.1371/journal.pgen.1004185)
Supplement: Table S3 — Genome wide sequence coverage data for samples used in this study. (PDF) [file pgen.1004185.s014.pdf]

		Without Oseltamivir		With Oseltamivir	
	**Passage**	**Median Coverage**	**Sites with Coverage < 100**	**Median Coverage**	**Sites with Coverage < 100**
**Replicate 1**	1	20590	2	20590	2
	2	18591	1	18591	1
	3	43939	2	43939	2
	4	73723	3	127428	2
	5	77750	3	77115	3
	6	69206	3	56423	3
	7	8023	12	2626	21
	8	3512	15	3065	34
	9	111131	3	104076	2
	10	133980	3	97879	3
	11	154481	2	127936	2
	12	125989	3	166521	3
**Replicate 2**	1	20590	2	20590	2
	2	18591	1	18591	1
	3	43939	2	43939	2
	4	179219	10	83916	2
	5	168799	3	78986	4
	6	183300	3	101664	3
	7	172172	3	45126	4
	8	71870	3	47918	3
	9	11449	5	9607	9
	10	10680	3	9496	3
	11	12649	2	8492	10
	12	15485	2	7988	19
